# Supplementary material for: Research on feature selection for AC contactor vibration signals based on regularized random forest with recursive selection
Source: PLoS One. 2024 Sep 6;19(9):e0310110. doi: 10.1371/journal.pone.0310110 (PMC11379156; doi:10.1371/journal.pone.0310110)
Supplement: Attachment — Submitted filename: Response to reviewer.docx [file pone.0310110.s001.docx]

Dear Editor and Reviewers,

On behalf of all the co-authors, I would like to extend our sincere gratitude for the invaluable and constructive comments provided on our manuscript entitled "Time-frequency domain feature selection method based on AC contactor vibration signal" (Manuscript ID: PONE-D-24-07820). We truly appreciate the time and effort invested by you, which has significantly contributed to enhancing the quality and depth of our research work.

Your insightful feedback has not only guided us in thoroughly revising the original manuscript but also encouraged us to meticulously include additional figures and data materials. We have made every effort to ensure that our findings are presented with more rigor and persuasiveness. We are confident that these revisions and additions will greatly improve the quality and clarity of our paper for the readers.

To clearly demonstrate our attentiveness to and response to each of your comments, we have employed a system of color-coding within the manuscript. We have used red fonts to highlight our direct responses to your comments and blue fonts to indicate the content that has been revised or added. We hope that this color-coding will facilitate your identification of our improvements and assist you in conducting a more efficient re-review.

We have made the following key revisions to our manuscript:

**1.Abstract Enhancement:** We have included more detailed descriptions of the datasets and experimental outcomes to provide a clearer understanding of our study's significance and validity.

**2.Introduction Strengthening:** We have emphasized the novel aspects of our research by providing a clearer distinction from existing studies.

**3.Theoretical Section Expansion:** We have introduced additional figures (Figs 1 and 2) to aid in the comprehension of complex theoretical concepts.

**4.Experiment and Data Processing Details:** We have detailed the experimental setup and the model of the vibration sensor used, along with a thorough explanation of our data processing procedures.

**5.Feature Selection and Analysis Clarification:** We have provided a more in-depth explanation of the relationship between the selected features and the vibration patterns observed in AC contactors.

**6.Redundancy Verification and Method Design Discussion:** We have discussed the limitations of existing feature selection methods and how our approach addresses these issues.

**7.Result Comparison and Analysis Enhancement:** We have included a comparative analysis of our RFRS method with other methods, elucidating its superiority.

**8.Conclusion Expansion:** We have expanded the conclusion section to more comprehensively discuss the practical implications and potential directions for future research.

**9.Writing Quality and Structural Adjustments:** We have refined the manuscript's writing style, reduced the use of technical jargon, and improved the overall structure for better readability.

**10.Universality of Experimental Setup:** We have clarified the representative nature of our experimental conditions and their alignment with real-world applications.

**11.Presentation of Figures and Tables:** We have standardized the presentation of our figures and tables for improved clarity and consistency.

**12.Technical Terminology Reduction:** We have minimized the use of technical terms to make the paper more accessible to a broader audience.

We eagerly await your further feedback and remain committed to doing our utmost to continue enhancing the quality of our manuscript. We kindly request that you review the revised version and provide any additional suggestions or queries you may have. We are honored to collaborate with you in advancing the frontiers of scientific research.

We thank you once again for your consideration and support.

Sincerely,

The Authors

**Reviewer #1:**

**Comment 1:**In the Abstract, specific datasets and experimental results can be added so that the reader can quickly understand the importance and validity of the study.
**Response:**

We are grateful for your constructive suggestion, which has reminded us of the critical role of the abstract in encapsulating the essence of our manuscript and highlighting the key outcomes of our work. This ensures that readers can promptly discern the significance and validity of our research findings.In response to your feedback, we have revised the abstract to provide a more comprehensive overview of the study and to prominently feature our main results.

The updated abstract now appears on the first page of our latest manuscript, and the modifications are as follows:

To address the redundancy of time-frequency domain features in the vibration signals of AC contactors and enhance the accuracy of their condition identification, this study innovatively introduces a feature selection strategy based on Regularized Random Forest with Recursive Selection (RFRS). In this research, we constructed a specialized vibration signal experimental platform for precise extraction of both time-domain and frequency-domain features of the AC contactor's vibration signals. By incorporating a regularization term into the random forest algorithm and integrating the concept of recursive feature elimination, we optimized the feature selection process, enabling the decision trees to more accurately split towards features with greater information gain. Compared to the ineffective feature reduction of traditional random forest algorithms, RFRS has demonstrated its significant superiority. In terms of identifying the aging state of contactors, the performance of RFRS is particularly prominent, with accuracy rates improved by 5.31% and 1.85% over the Spearman's Rho method and the Filter method, respectively. This achievement not only enhances the accuracy of AC contactor condition identification but also provides strong theoretical support and practical guidance for the development of fault diagnosis and life prediction technologies for similar equipment in power systems.

**Comment 2:**In the Introduction section, differences and innovations from existing research can be more prominently emphasized.

**Response:**

Thank you for your valuable suggestion, which has made us aware of the shortcomings in emphasizing the innovation of our research within the introduction section. We have made corresponding revisions to this part to more clearly highlight the novelty and innovative contributions of our study.

These revisions are reflected in the introduction section, spanning from the first to the third page of our latest manuscript. Due to space limitations, we have only listed some of the main content that has been modified. Below is a summary of the main modifications we have made:

......Currently, most scholarly research on the state recognition of AC contactors primarily focuses on electrical and mechanical parameters. In reference [4], five parameters including contact resistance and closing time are studied. The remaining electrical life of the contacts is predicted using Pearson's correlation coefficient method and LSTM neural network, achieving an accuracy rate of over 93%. Reference [5] employs the Wiener method to statistically analyze the quality loss data of the contacts at different stages, predicting the remaining life of the contacts with an error of less than 10%. Reference [6] utilizes an autoencoder neural network to compress and integrate electrical and mechanical parameters, extracting a comprehensive health indicator that characterizes the contact state, which enhances robustness by 5% compared to traditional methods. References [7-10] implement predictions of the remaining life of the contacts using various neural network algorithms. Reference [11] pre-processes the electrical and mechanical parameters into high-dimensional data and applies principal component analysis to reduce the dimensionality of this data. Subsequently, based on subjective experience, important components are selected as inputs for PSO-LSSVM to more accurately identify the degradation state of AC contactors on small sample datasets. Reference [12] establishes an audio model of the contactor during the making process and predicts the remaining life of the contactor with a lower error method combined with CNN algorithm. The aforementioned studies concentrate on introducing new algorithms into traditional datasets without conducting a detailed study on the vibration signals of AC contactors, and have yet to determine the key features that best represent the state of the contactors. In contrast, there is a certain consensus among researchers on the use of vibration signals for critical power equipment, such as employing time-domain amplitude and frequency-domain amplitude as key features for identifying the operating state of high-voltage circuit breakers [13]. This consensus aids subsequent researchers in reducing the scale of data and enhancing model recognition accuracy. Therefore, it is of great importance to conduct an in-depth study of the variation patterns of AC contactor vibration signals and to identify the time-domain and frequency-domain features that can best represent the changes in the state of the contactors.

Statistical analysis of the time-domain waveform and frequency-domain waveform of vibration signals can yield high-dimensional feature data, such as amplitude, amplitude standard deviation, and amplitude variance. It is known from statistical knowledge that the standard deviation is closely related to variance, implying potential redundancy in high-dimensional features where multiple features may be linearly correlated, thus leading to reduced accuracy or efficiency of the state recognition model. Mainstream feature selection methods include the correlation coefficient method, embedded method, filter method, and recursive elimination method. The correlation coefficient method is relatively straightforward, but its threshold setting is subjective, requiring multiple judgments to select optimal features, resulting in low efficiency for high-dimensional datasets [14-18]. The embedded method is suitable for high-dimensional datasets, often using the random forest model as the base learner. Compared to the correlation coefficient method, the embedded method significantly improves feature selection efficiency. However, due to its unbiased nature, the embedded method may split nodes along directions of non-critical features, affecting the model's decision-making process [19-27]. The recursive elimination method exhaustively searches all possible feature combinations, which is beneficial for identifying true key features, but it is time-consuming on high-dimensional datasets and dependent on the type of base learner. Therefore, this paper introduces a regularization term into the random forest, biasing it and reducing consideration of unimportant features. Concurrently, this paper integrates the improved random forest with the concept of recursive elimination, addressing the dependency of the recursive elimination method on the base learner type, and combines different features based on feature importance, solving the time-consuming issue of enumeration and efficiently selecting key features......

**Comment 3:In the Basic theory section, examples or diagrams can be added to help readers better understand complex concepts.**

**Response:**

Thank you for your suggestion. We have added multiple figures to the Fundamental Theory section to ensure that readers can better understand the complex concepts as well as the innovative aspects of our paper.

The revised content includes Fig 1 on page 3 and Fig 2 on page 5. The modifications are as follows:

Fig 1. Principle diagram of the random forest algorithm

Fig. 2 Principle diagram of regularized recursive selection random forest

**Comment 4:The Experiment and Data Processing section should detail the data preprocessing steps to ensure reproducible results.
Response:**

Thank you for your constructive feedback. In the revised manuscript, we have included a schematic diagram of the experiment to provide a clearer visual representation of our experimental setup. Additionally, we have specified the model of the vibration sensor used in our study. Furthermore, we have elaborated on the data processing procedure, which encompasses the methodology for feature extraction and the determination of sample labels.

These revisions can be found in Section 3, spanning from pages 6 to 10 of the manuscript. Due to space limitations, we are providing a summary of some of the key modifications below:

**3.3.2 Feature Extraction**

When samples are entered into the offline database, accurate classification of the samples is crucial for subsequent data analysis or decision-making activities. This paper, taking the CJX2-5011 model AC contactor as an example, elaborates on the criteria for sample classification.

In Section 2.1, the working principle and failure mechanisms of AC contactors are described in detail. Research indicates that the failures of AC contactors are primarily divided into two types: welding and mechanical interlocking. Although these two failure phenomena have different manifestations, they are consistent in physical essence (there is a deviation in the relative position of the moving and static contacts). As shown in Fig 3, the kinematic manifestation of the welding phenomenon is that after the moving and static contacts are engaged, the moving contact continues to move forward for a certain distance, a process that can be characterized by overtravel. The kinematic manifestation of the mechanical interlocking phenomenon is that after the moving and static contacts are engaged, they cannot separate normally, a process that can be characterized by the minimum distance between the moving and static contacts (opening distance). By measuring the relative position between the moving and static contacts, the current working state of the contacts can be determined.

Fig 5 demonstrates the trends in overtravel and opening distance variation across the entire operational lifespan of the contactor. The change in opening distance shows a periodic characteristic, evident in three distinct stages: Stage I (with the number of operations ranging from 10,000 to 30,000 times), Stage II (with the number of operations ranging from 30,000 to 70,000 times), and Stage III (with the number of operations ranging from 70,000 to 90,000 times), where peak phenomena are

**Fig.5 Trends in overtravel and opening distance variations**

observed in each stage. Compared to the opening distance, the variation in overtravel exhibits a stepwise pattern, aligning well with the irreversible nature of contact aging. During Stage I (with the number of operations ranging from 10,000 to 40,000 times), the amplitude change of overtravel is not pronounced; after Stage II (with the number of operations ranging from 40,000 to 70,000 times), there is a marked increase in the amplitude of overtravel; and in Stage III (with the number of operations ranging from 70,000 to 90,000 times), the rate of increase in the amplitude of overtravel accelerates. Based on the above analysis, this study selects the change in overtravel as the primary criterion for classifying samples......

**Comment 5:The Feature selection and analysis section can add background information on the comparison methods so that readers can understand the principles and differences between the methods.**

**Response:**

Thank you for your insightful suggestion. In the latest manuscript, to elucidate the distinctions between our method and other feature selection methodologies, we have made the following enhancements:

(1)Introduction Section: In the introduction (p. 2), we have succinctly introduced the advantages and limitations of various feature selection methods. This brief overview provides a foundation for understanding the context of our work within the existing literature:

......Statistical analysis of the time-domain waveform and frequency-domain waveform of vibration signals can yield high-dimensional feature data, such as amplitude, amplitude standard deviation, and amplitude variance. It is known from statistical knowledge that the standard deviation is closely related to variance, implying potential redundancy in high-dimensional features where multiple features may be linearly correlated, thus leading to reduced accuracy or efficiency of the state recognition model. Mainstream feature selection methods include the correlation coefficient method, embedded method, filter method, and recursive elimination method. The correlation coefficient method is relatively straightforward, but its threshold setting is subjective, requiring multiple judgments to select optimal features, resulting in low efficiency for high-dimensional datasets [14-18]. The embedded method is suitable for high-dimensional datasets, often using the random forest model as the base learner. Compared to the correlation coefficient method, the embedded method significantly improves feature selection efficiency. However, due to its unbiased nature, the embedded method may split nodes along directions of non-critical features, affecting the model's decision-making process [19-27]. The recursive elimination method exhaustively searches all possible feature combinations, which is beneficial for identifying true key features, but it is time-consuming on high-dimensional datasets and dependent on the type of base learner. Therefore, this paper introduces a regularization term into the random forest, biasing it and reducing consideration of unimportant features. Concurrently, this paper integrates the improved random forest with the concept of recursive elimination, addressing the dependency of the recursive elimination method on the base learner type, and combines different features based on feature importance, solving the time-consuming issue of enumeration and efficiently selecting key features......

(2)Results and Analysis Section: We have included a detailed comparison of our feature selection method with other existing methods in Section 4.3, which spans from pages 13 to 15. This detailed description aims to highlight the unique aspects of our approach and how it differs from and potentially improves upon other methods:

**4.3 Results and Analysis**

In Section 3.2, this study detailed the specific application of Spearman's rho for the selection of vibration features in AC contactors. The Filter method is similar and, therefore, the implementation process of the Filter method is not further elaborated. The embedding method is akin to RFRS, yet it differs in that it employs a traditional Random Forest (RF) algorithm without adjustments or optimizations. The outcomes of the four feature selection methods are presented in Table 3.

It is evident from Table 3 that the embedding method fails to effectively select key features. This is attributed to the RF's unbiased node splitting direction, designed to accommodate various tasks, which results in an overemphasis on minor gain directions and an inability to utilize known information to guide the splitting direction. In contrast, RFRS and the other two feature selection methods have efficiently reduced the feature scale, and they collectively identify T1 (time-domain peak-to-peak amplitude), T5 (time-domain amplitude standard deviation), and T7 (time-domain skewness factor) as the most critical vibration features. However, it should be noted that there are distinct differences in the decision-making for other key features among these three feature selection methods: RFRS considers T3, T6, and F6 as key features; Spearman's rho regards T6 and F6 as key features; and the Filter method identifies F2 and F10 as key features. To evaluate the merits of these three feature selection methods, this study assesses the features using four common classifiers, with the metrics of recall rate, F1-Score, and precision. The corresponding results are depicted in Table 4......

****Comment 6:**The Redundancy verification and comparison method design section can further discuss the reasons for redundancy between features and how future research can avoid it.**

**Response:**

Thank you for your valuable suggestions. At present, in the research on electrical equipment such as high-voltage circuit breakers, there is a consensus within the academic community that key features for fault diagnosis studies should include the amplitude of time-domain signals, among other things. In addition to this, there are other areas of agreement. However, the application of vibration signals to the field of AC contactors is less explored, which is the primary motivation for our study.

During the process of feature extraction from vibration signals, we observed that some features exhibit statistical redundancy or correlation, while others are not directly derived from the vibration signals but are instead obtained through mathematical operations on other features, contributing to feature redundancy. We have designed and compared different feature selection methods to demonstrate to our readers the existence and importance of the feature redundancy issue. This also serves to highlight that traditional feature selection methods are often overly dependent on subjective experience. Moreover, traditional methods may become ineffective or inefficient when dealing with high-dimensional feature sets.

In our future research, we plan to continue expanding our database and to explore more advanced feature extraction techniques to further enhance the depth and breadth of our study.

This part of the content has been supplemented in the following sections of our manuscript: the Introduction, Section 3.1 titled "Feature Selection Model Based on RFRS," and Section 4.3 titled "Results and Analysis," for example:

......Statistical analysis of the time-domain waveform and frequency-domain waveform of vibration signals can yield high-dimensional feature data, such as amplitude, amplitude standard deviation, and amplitude variance. It is known from statistical knowledge that the standard deviation is closely related to variance, implying potential redundancy in high-dimensional features where multiple features may be linearly correlated, thus leading to reduced accuracy or efficiency of the state recognition model. Mainstream feature selection methods include the correlation coefficient method, embedded method, filter method, and recursive elimination method......

......It is evident from Table 3 that the embedding method fails to effectively select key features. This is attributed to the RF's unbiased node splitting direction, designed to accommodate various tasks, which results in an overemphasis on minor gain directions and an inability to utilize known information to guide the splitting direction......

**Comment 7:The Comparative results and analysis section explains why the BO-SGRRF method is superior to other methods.**

**Response:**

Thank you for your affirmation of our work! In the latest revised manuscript, we have made significant changes to the article based on your feedback to ensure a better understanding of our work by you and our readers.

**Comment 8:The Conclusion section can further discuss the implications of the results for practical applications and potential directions for future research.**

**Response:**

Thank you for your guidance and suggestions! With your help, the quality of our paper has been further enhanced. In the latest manuscript, we have expanded our discussion on the significance and potential future directions of applying vibration signals to the state recognition of AC contactors. Traditionally, researchers have relied on multiple voltage and current sensors for related studies. Our research, however, will enable future studies to be conducted using only a single vibration sensor for fault diagnosis and other related research.

We have made revisions to the conclusion section of our manuscript, which can now be found on pages 15 to 16 of the conclusion section. The revised content includes the following:

This study proposes a feature selection method based on Regularized Random Forest with Recursive Selection (RFRS) to address the issue of feature redundancy in the vibration signals of AC contactors. Through comprehensive analysis and experimental validation, the following conclusions have been drawn:

(1)The RFRS feature selection method introduced in this study effectively extracts key vibration features of AC contactors and reduces the dimensionality of the feature set, outperforming current mainstream feature selection methods.

(2)In the identification of the aging state of AC contactors (stages II and III), the performance of RFRS is 5.31% and 1.85% higher than that of Spearman's rho and the Filter method, respectively. Additionally, this study found that RFRS is more prone to classifying the normal state (stage I) as an aging state (stages II and III) compared to the two aforementioned methods. After considering these two characteristics comprehensively, the performance of RFRS is 0.31% and 1.83% higher than that of Spearman's rho and the Filter method, respectively. These results suggest that RFRS is generally superior to current mainstream feature selection methods, although the specific application scenarios still require researchers to make decisions based on actual needs.

(3)There remain numerous challenges in conducting state identification research on AC contactors from the perspective of vibration signals, particularly in how to extract features with more pronounced trends or more stable changes from the vibration signals. Therefore, future work will focus on two aspects: one is the continuous expansion of the database; the other is the improvement of data quality and the exploration of new feature extraction techniques. In summary, the RFRS-based feature selection method proposed in this study has demonstrated its effectiveness in resolving the issue of time-frequency domain feature redundancy in the vibration signals of AC contactors, compared to traditional methods.

In summary, the feature selection method based on Regularized Random Forest with Recursive Selection (RFRS) proposed in this study has demonstrated its effectiveness in addressing the issue of time-frequency domain feature redundancy in the vibration signals of AC contactors, showing superiority over traditional methods.

**Reviewer #2:**The paper appears to be focused on the application of machine learning techniques for the diagnosis of contactor degradation in electrical systems, Here are my comments:

We are grateful for the numerous comments provided on our manuscript, which have greatly assisted us in enhancing the overall quality of our work and brought to our attention several details that we had overlooked. In response to your feedback, we have made extensive revisions to the article, encompassing not only the diction and grammar but also the structure of the paper, the consistency of figures and tables, the specifics of our experimental procedures, and the presentation of results.

We will address all of your comments based on the latest version of the manuscript and sincerely request your constructive feedback on the revised draft.

Thank you once again for your valuable input.

**Comment 1:**The paper fails to clearly articulate the specific problem it aims to address within the domain of AC contactor vibration signal analysis. Without a well-defined problem statement, the significance and relevance of the proposed method remain obscure.

**Response:**

Thank you for your feedback! We have revised all sections of the article that describe the issues, with particular attention given to the Introduction. In the Introduction, we have articulated the current challenges in research related to AC contactors, specifically the insufficient utilization of vibration signals. In fault diagnosis studies for equipment such as high-voltage circuit breakers, researchers have established that the time-domain amplitude of vibration signals is the most critical feature and have also identified the specific frequencies of impact signals during fault occurrences. However, for AC contactors, a consensus on these aspects has not yet been reached. The impetus for our work is to discuss the application of vibration signals within AC contactors. Upon conducting relevant experiments, we discovered that the dimensionality of the vibration feature set is high, and mainstream feature selection methods are not capable of efficiently selecting key features. Therefore, this study was undertaken to address the high redundancy of vibration features in AC contactors.

The updated content can be found in the Introduction section, spanning pages 1 to 3 of the manuscript.

Under the backdrop of the "dual carbon" strategic goals, the construction and development of a new type of power system must achieve higher efficiency in the utilization of energy and electrical equipment, extend the service life of the equipment, and more accurately grasp the operating state and remaining life of the equipment [1-3]. Compared to high-voltage equipment such as circuit breakers, AC contactors have a simpler structure, relying solely on air for arc extinguishing, and are widely used in situations where the circuit is frequently opened and closed. The state of an AC contactor is mainly determined by the moving and static contacts. The action of the contacts coming together allows the current to flow normally through the circuit, while their separation interrupts the current. In a more enclosed environment, factors such as contact surface wear and the thermal effect of the current reduce the breakdown voltage of the air medium. As the number of contact operations increases, an electric arc is generated between the moving and static contacts. Due to the poor air circulation, the arc is not easily extinguished, continuously eroding the contacts, damaging the surface morphology, and even fusing the moving and static contacts together, preventing the circuit from being cut off and causing the circuit to become uncontrollable. Therefore, accurately grasping the operating state of AC contactors can provide data support for the operation and maintenance of control equipment and ensure the stability and safety of the power system.

Currently, most scholarly research on the state recognition of AC contactors primarily focuses on electrical and mechanical parameters. In reference [4], five parameters including contact resistance and closing time are studied. The remaining electrical life of the contacts is predicted using Pearson's correlation coefficient method and LSTM neural network, achieving an accuracy rate of over 93%. Reference [5] employs the Wiener method to statistically analyze the quality loss data of the contacts at different stages, predicting the remaining life of the contacts with an error of less than 10%. Reference [6] utilizes an autoencoder neural network to compress and integrate electrical and mechanical parameters, extracting a comprehensive health indicator that characterizes the contact state, which enhances robustness by 5% compared to traditional methods. References [7-10] implement predictions of the remaining life of the contacts using various neural network algorithms. Reference [11] pre-processes the electrical and mechanical parameters into high-dimensional data and applies principal component analysis to reduce the dimensionality of this data. Subsequently, based on subjective experience, important components are selected as inputs for PSO-LSSVM to more accurately identify the degradation state of AC contactors on small sample datasets. Reference [12] establishes an audio model of the contactor during the making process and predicts the remaining life of the contactor with a lower error method combined with CNN algorithm. The aforementioned studies concentrate on introducing new algorithms into traditional datasets without conducting a detailed study on the vibration signals of AC contactors, and have yet to determine the key features that best represent the state of the contactors. In contrast, there is a certain consensus among researchers on the use of vibration signals for critical power equipment, such as employing time-domain amplitude and frequency-domain amplitude as key features for identifying the operating state of high-voltage circuit breakers [13]. This consensus aids subsequent researchers in reducing the scale of data and enhancing model recognition accuracy. Therefore, it is of great importance to conduct an in-depth study of the variation patterns of AC contactor vibration signals and to identify the time-domain and frequency-domain features that can best represent the changes in the state of the contactors.

Statistical analysis of the time-domain waveform and frequency-domain waveform of vibration signals can yield high-dimensional feature data, such as amplitude, amplitude standard deviation, and amplitude variance. It is known from statistical knowledge that the standard deviation is closely related to variance, implying potential redundancy in high-dimensional features where multiple features may be linearly correlated, thus leading to reduced accuracy or efficiency of the state recognition model. Mainstream feature selection methods include the correlation coefficient method, embedded method, filter method, and recursive elimination method. The correlation coefficient method is relatively straightforward, but its threshold setting is subjective, requiring multiple judgments to select optimal features, resulting in low efficiency for high-dimensional datasets [14-18]. The embedded method is suitable for high-dimensional datasets, often using the random forest model as the base learner. Compared to the correlation coefficient method, the embedded method significantly improves feature selection efficiency. However, due to its unbiased nature, the embedded method may split nodes along directions of non-critical features, affecting the model's decision-making process [19-27]. The recursive elimination method exhaustively searches all possible feature combinations, which is beneficial for identifying true key features, but it is time-consuming on high-dimensional datasets and dependent on the type of base learner. Therefore, this paper introduces a regularization term into the random forest, biasing it and reducing consideration of unimportant features. Concurrently, this paper integrates the improved random forest with the concept of recursive elimination, addressing the dependency of the recursive elimination method on the base learner type, and combines different features based on feature importance, solving the time-consuming issue of enumeration and efficiently selecting key features.

In summary, to further explore the application of vibration signals in the field of state recognition for AC contactors and to address the issue of high redundancy in both time-domain and frequency-domain features of these signals, this paper proposes a vibration feature selection method for AC contactors based on Regularized Random Forest with Recursive Selection (RFRS). Firstly, this study constructs an experimental platform for AC contactors based on vibration signals, in accordance with relevant testing standards. Subsequently, the platform is utilized to extract both time-domain and frequency-domain features of the vibration signals. Furthermore, by introducing a regularization term and integrating the concept of recursive feature elimination, improvements are made to the random forest algorithm, enabling it to effectively address the selection of time-frequency domain features from AC contactor vibration signals.

**Comment 2:**The proposed feature selection method, BO-SGRRF, appears convoluted and unnecessarily intricate. The integration of Bayesian Optimization, Guided Regularized Random Forest (GRRF), and Stepwise selection introduces unnecessary complexity without clear justification for their combined usage.

**Response:**

Thank you for your valuable feedback! Initially, we introduced Bayesian optimization with the aim of enhancing the model's adaptability and ensuring optimal performance when processing data from an updated database. However, after conducting relevant experiments, we realized that Bayesian optimization indeed complicates the model, which already possesses self-optimization capabilities and does not necessitate further optimization. Your constructive comments have been extremely helpful to us! In our latest research, we have integrated a random forest, a regularization term, and stepwise selection, which has helped us effectively select key features.

(1)We introduced a regularization term into the random forest, which differs from traditional methods. Traditionally, regularization is incorporated into feature weights, whereas we introduced the regularization term during the node splitting of decision trees, fully leveraging known information to guide the split. In the revised manuscript, on pages 14 to 15, we compared this integrated model with a traditional random forest that did not incorporate this approach. The results indicate that our integrated model outperforms the traditional random forest.

(2)We integrated stepwise selection into our model because we aimed to identify an optimal feature combination that would not be adversely affected by the accuracy rate of any single state, ensuring that the overall accuracy rate is not influenced by outliers. Another reason for integrating stepwise selection is that, for high-dimensional features, random combinations do not guarantee that researchers will find the optimal solution, and enumeration methods would reduce efficiency.

In Section 3.2, this study detailed the specific application of Spearman's rho for the selection of vibration features in AC contactors. The Filter method is similar and, therefore, the implementation process of the Filter method is not further elaborated. The embedding method is akin to RFRS, yet it differs in that it employs a traditional Random Forest (RF) algorithm without adjustments or optimizations. The outcomes of the four feature selection methods are presented in Table 3.

It is evident from Table 3 that the embedding method fails to effectively select key features. This is attributed to the RF's unbiased node splitting direction, designed to accommodate various tasks, which results in an overemphasis on minor gain directions and an inability to utilize known information to guide the splitting direction. In contrast, RFRS and the other two feature selection methods have efficiently reduced the feature scale, and they collectively identify T1 (time-domain peak-to-peak amplitude), T5 (time-domain amplitude standard deviation), and T7 (time-domain skewness factor) as the most critical vibration features. However, it should be noted that there are distinct differences in the decision-making for other key features among these three feature selection methods: RFRS considers T3, T6, and F6 as key features; Spearman's rho regards T6 and F6 as key features; and the Filter method identifies F2 and F10 as key features. To evaluate the merits of these three feature selection methods, this study assesses the features using four common classifiers, with the metrics of recall rate, F1-Score, and precision. The corresponding results are depicted in Table 4.

**Table 3　Results of feature selection**

| Feature selection method | Feature set | Selection result |
| --- | --- | --- |
| RFRS | T1-T7  F1-F10 | T1,T3,T5,T6,T7,F6 |
| Spearman's Rho |  | T1,T5,T6,T7,F6 |
| Embedded |  | T1-T7  F1-F10 |
| Filter |  | T1,T5,T7,F2,F10 |

**Table 4　Performance of different feature selection methods on classifiers**

| Feature selection method | Classifier | Recall | F1-Score | Precision |
| --- | --- | --- | --- | --- |
| RFRS | LGBM | 90.78% | 90.72% | 91.70% |
|  | KNN | 87.51% | 87.56% | 88.65% |
|  | SVM | 87.49% | 87.57% | 88.47% |
|  | LR | 83.69% | 83.78% | 84.71% |
| Spearman's Rho | LGBM | 90.18% | 90.14% | 91.09% |
|  | KNN | 86.67% | 86.73% | 87.56% |
|  | SVM | 87.27% | 87.33% | 88.17% |
|  | LR | 84.12% | 84.20% | 85.40% |
| Embedded | LGBM | 88.69% | 88.66% | 89.92% |
|  | KNN | 83.78% | 83.91% | 84.88% |
|  | SVM | 87.07% | 87.15% | 87.82% |
|  | LR | 83.77% | 83.83% | 84.48% |
| Filter | LGBM | 87.29% | 87.35% | 87.98% |
|  | KNN | 85.11% | 85.19% | 85.82% |
|  | SVM | 86.72% | 86.76% | 87.27% |
|  | LR | 82.95% | 83.0% | 83.86% |

The results from the classifiers reveal that the Embedded method, despite incorporating all features as inputs to the model, yields lower scores across the three metrics compared to the other three feature selection methods. This observation suggests that there is not always a positive correlation between the number of features and model performance; in fact, an increased number of features can often lead to a decrease in model performance. Consequently, the selection of features prior to data analysis or decision-making is a crucial measure to ensure the efficacy of the research.

As depicted in Table 4, the average scores for Recall, F1-Score, and Precision for RFRS are 87.37%, 87.41%, and 84.71%, respectively; for Spearman's rho, the corresponding scores are 82.06%, 87.1%, and 88.06%, respectively; and for the Filter method, the scores are 85.52%, 85.58%, and 86.23%, respectively. Comparatively, the Recall score of RFRS is 5.31% higher than that of Spearman's rho and 1.85% higher than the Filter method; its F1-Score is 0.31% higher than Spearman's rho and 1.83% higher than the Filter method; whereas its Precision score is 3.35% lower than Spearman's rho and 1.52% lower than the Filter method. These outcomes indicate that RFRS surpasses Spearman's rho, the Filter method, and the Embedded method in the state identification of stages II and III of AC contactors. Conversely, in the state identification of stage I, RFRS is less effective than Spearman's rho and the Filter method, as it may erroneously classify stage I samples as belonging to stages II and III, which could result in premature alerts in practical scenarios. However, when considering the F1-Score—a comprehensive metric that encapsulates both Recall and Precision—RFRS is favored over the other feature selection methodologies.

**Comment 3:The paper lacks a thorough explanation of why the selected methodologies (BO, GRRF, Stepwise selection) are most suitable for addressing the identified problem. Without robust justification, the credibility and effectiveness of the proposed approach are questionable.**

**Response:**

Thank you for your comments! To enhance the credibility and effectiveness of the method proposed in our study, we have provided a brief overview of the limitations of current feature selection methods and introduced the concept of our method in the Introduction section, spanning pages 1 to 3 of the manuscript. Additionally, in the Results section, from pages 13 to 15, we have demonstrated that the method presented in this paper is more effective than current feature selection methods.

......Statistical analysis of the time-domain waveform and frequency-domain waveform of vibration signals can yield high-dimensional feature data, such as amplitude, amplitude standard deviation, and amplitude variance. It is known from statistical knowledge that the standard deviation is closely related to variance, implying potential redundancy in high-dimensional features where multiple features may be linearly correlated, thus leading to reduced accuracy or efficiency of the state recognition model. Mainstream feature selection methods include the correlation coefficient method, embedded method, filter method, and recursive elimination method. The correlation coefficient method is relatively straightforward, but its threshold setting is subjective, requiring multiple judgments to select optimal features, resulting in low efficiency for high-dimensional datasets [14-18]. The embedded method is suitable for high-dimensional datasets, often using the random forest model as the base learner. Compared to the correlation coefficient method, the embedded method significantly improves feature selection efficiency. However, due to its unbiased nature, the embedded method may split nodes along directions of non-critical features, affecting the model's decision-making process [19-27]. The recursive elimination method exhaustively searches all possible feature combinations, which is beneficial for identifying true key features, but it is time-consuming on high-dimensional datasets and dependent on the type of base learner. Therefore, this paper introduces a regularization term into the random forest, biasing it and reducing consideration of unimportant features. Concurrently, this paper integrates the improved random forest with the concept of recursive elimination, addressing the dependency of the recursive elimination method on the base learner type, and combines different features based on feature importance, solving the time-consuming issue of enumeration and efficiently selecting key features......

**Comment 4:**The writing style of the paper is dense and jargon-heavy, making it difficult for readers to grasp the key concepts and contributions of the research. The lack of clarity in presentation impedes the understanding and evaluation of the proposed method.

**Response:**

Thank you for your feedback. The root cause of the issue lies in the fact that the authors' native language is not English, but we understand that this cannot be considered a sufficient excuse. In order to facilitate a better understanding of our work by the readers, we have made revisions in the manuscript to reduce the use of technical jargon and have included examples or figures to aid in the comprehension of the concepts. We have sought assistance from researchers with extensive experience in English writing and have rewritten the paper based on their suggestions. We sincerely invite you to provide your comments on the revised manuscript. We are genuinely looking forward to your response!

**Comment 5:**The paper overlooks potential limitations and drawbacks of the proposed method, such as computational complexity, sensitivity to parameter tuning, and generalizability to diverse datasets. Ignoring these limitations undermines the credibility and reliability of the research findings.

**Response:**

First and foremost, we extend our heartfelt gratitude for the invaluable comments and suggestions you have provided. The potential limitations and drawbacks of our proposed method, as pointed out by you, including computational complexity, sensitivity to parameter tuning, and generalizability across diverse datasets, are extremely pertinent and serve as a significant guide for our work.

Upon earnest consideration of your feedback, we undertook a series of experiments and debugging processes. We discovered that the computational complexity is predominantly influenced by the Bayesian optimization and the choice of the base learner. In particular, the number of iterations and the surrogate model parameters in Bayesian optimization indeed led to extended computational times and excessive sensitivity to parameters. After thorough analysis and multiple rounds of debugging, we observed that our proposed method meets the performance requirements even without the integration of an optimization model. With this insight, we have excised the section pertaining to Bayesian optimization from the latest revision of the manuscript.

Furthermore, we have noted that the type of base learner also significantly impacts computational complexity. For instance, models such as Support Vector Machines and k-Nearest Neighbors may require hundreds of seconds to perform state recognition tasks, whereas tree-based models can accomplish these tasks within seconds. We have added a comparative analysis of various common classifiers on pages 14 to 15 of the manuscript to demonstrate the superiority of our method.

Lastly, we would like to emphasize that this study is dedicated to the application of vibration signals in AC contactors, with the dataset used being derived from actual experimental data. We are continuously conducting experiments to further expand and enrich our database.

We have augmented the revised manuscript with a detailed discussion of these limitations to enhance the transparency and credibility of our research. We look forward to any further feedback and sincerely hope to receive your valuable comments and suggestions.

**Comment 6:**The paper fails to discuss the practical implications and real-world applications of the proposed method. Without addressing how the research findings can be applied in practical settings, its relevance and impact remain uncertain.

**Response:**

Thank you for your feedback! We would like to clarify that we have constructed an experimental platform based on vibration signals for AC contactors. To ensure the reproducibility of our experiments, we have detailed numerous experimental details and data processing methods on pages 6 to 10 of the latest manuscript. All the data we used were derived from actual physical experiments, not from program simulations. Furthermore, our experimental conditions were set according to Chinese national standards, which every AC contactor must adhere to during actual operation. Therefore, our experimental data closely approximates the conditions in real-world applications.

**Comment 7:**The paper relies heavily on previous research results and experimental data without introducing significant novel contributions to the field. It merely replicates existing methodologies without advancing the state of the art.

**Response:**

Thank you for your feedback! We understand and appreciate your concerns. In our previous drafts, we listed past research findings and based our work on those results in an effort to enhance the persuasiveness of our paper. However, based on your comments, we realized that this approach could lead to misunderstandings among our readers. Therefore, we have rewritten the paper and included clear diagrams to illustrate the innovations of our work.

Not only have we established an experimental platform based on vibration signals (with experimental details and data processing methods thoroughly disclosed in the manuscript), but we have also introduced a feature selection method based on Regularized Random Forest with Recursive Selection. This method demonstrates superior performance compared to current mainstream feature selection methods (specific comparison results can be found on pages 14 to 15 of the manuscript).

**4.3 Results and Analysis**

In Section 3.2, this study detailed the specific application of Spearman's rho for the selection of vibration features in AC contactors. The Filter method is similar and, therefore, the implementation process of the Filter method is not further elaborated. The embedding method is akin to RFRS, yet it differs in that it employs a traditional Random Forest (RF) algorithm without adjustments or optimizations. The outcomes of the four feature selection methods are presented in Table 3.

It is evident from Table 3 that the embedding method fails to effectively select key features. This is attributed to the RF's unbiased node splitting direction, designed to accommodate various tasks, which results in an overemphasis on minor gain directions and an inability to utilize known information to guide the splitting direction. In contrast, RFRS and the other two feature selection methods have efficiently reduced the feature scale, and they collectively identify T1 (time-domain peak-to-peak amplitude), T5 (time-domain amplitude standard deviation), and T7 (time-domain skewness factor) as the most critical vibration features. However, it should be noted that there are distinct differences in the decision-making for other key features among these three feature selection methods: RFRS considers T3, T6, and F6 as key features; Spearman's rho regards T6 and F6 as key features; and the Filter method identifies F2 and F10 as key features. To evaluate the merits of these three feature selection methods, this study assesses the features using four common classifiers, with the metrics of recall rate, F1-Score, and precision. The corresponding results are depicted in Table 4.

**Table 3　Results of feature selection**

| Feature selection method | Feature set | Selection result |
| --- | --- | --- |
| RFRS | T1-T7  F1-F10 | T1,T3,T5,T6,T7,F6 |
| Spearman's Rho |  | T1,T5,T6,T7,F6 |
| Embedded |  | T1-T7  F1-F10 |
| Filter |  | T1,T5,T7,F2,F10 |

**Table 4　Performance of different feature selection methods on classifiers**

| Feature selection method | Classifier | Recall | F1-Score | Precision |
| --- | --- | --- | --- | --- |
| RFRS | LGBM | 90.78% | 90.72% | 91.70% |
|  | KNN | 87.51% | 87.56% | 88.65% |
|  | SVM | 87.49% | 87.57% | 88.47% |
|  | LR | 83.69% | 83.78% | 84.71% |
| Spearman's Rho | LGBM | 90.18% | 90.14% | 91.09% |
|  | KNN | 86.67% | 86.73% | 87.56% |
|  | SVM | 87.27% | 87.33% | 88.17% |
|  | LR | 84.12% | 84.20% | 85.40% |
| Embedded | LGBM | 88.69% | 88.66% | 89.92% |
|  | KNN | 83.78% | 83.91% | 84.88% |
|  | SVM | 87.07% | 87.15% | 87.82% |
|  | LR | 83.77% | 83.83% | 84.48% |
| Filter | LGBM | 87.29% | 87.35% | 87.98% |
|  | KNN | 85.11% | 85.19% | 85.82% |
|  | SVM | 86.72% | 86.76% | 87.27% |
|  | LR | 82.95% | 83.0% | 83.86% |

The results from the classifiers reveal that the Embedded method, despite incorporating all features as inputs to the model, yields lower scores across the three metrics compared to the other three feature selection methods. This observation suggests that there is not always a positive correlation between the number of features and model performance; in fact, an increased number of features can often lead to a decrease in model performance. Consequently, the selection of features prior to data analysis or decision-making is a crucial measure to ensure the efficacy of the research.

As depicted in Table 4, the average scores for Recall, F1-Score, and Precision for RFRS are 87.37%, 87.41%, and 84.71%, respectively; for Spearman's rho, the corresponding scores are 82.06%, 87.1%, and 88.06%, respectively; and for the Filter method, the scores are 85.52%, 85.58%, and 86.23%, respectively. Comparatively, the Recall score of RFRS is 5.31% higher than that of Spearman's rho and 1.85% higher than the Filter method; its F1-Score is 0.31% higher than Spearman's rho and 1.83% higher than the Filter method; whereas its Precision score is 3.35% lower than Spearman's rho and 1.52% lower than the Filter method. These outcomes indicate that RFRS surpasses Spearman's rho, the Filter method, and the Embedded method in the state identification of stages II and III of AC contactors. Conversely, in the state identification of stage I, RFRS is less effective than Spearman's rho and the Filter method, as it may erroneously classify stage I samples as belonging to stages II and III, which could result in premature alerts in practical scenarios. However, when considering the F1-Score—a comprehensive metric that encapsulates both Recall and Precision—RFRS is favored over the other feature selection methodologies.

**Comment 8:**While the paper outlines the experimental conditions and data processing methods, it lacks sufficient detail on the experimental setup, data collection procedures, and data analysis techniques. This lack of transparency makes it difficult for readers to replicate the study or assess the validity of the results.

**Response:**

Thank you for your insightful feedback! We have taken care to provide detailed descriptions of the experimental procedures and data processing techniques in our latest manuscript. This is to ensure that our study can be effectively replicated and its results critically evaluated by readers.

**3.2 Experimental Conditions**

Fig 4(a) illustrates the experimental platform for the vibration signals of AC contactors. The platform employs a KS76C100 single-axis accelerometer to measure the vibration signals. This sensor has an acceleration range of 60g and an output signal of 100mV/g, which is capable of undertaking a variety of vibration measurements. Additionally, the sensor is equipped with an IEPE (Integrated Electronics Piezoelectric) output function, allowing for a direct connection with a data acquisition card, which helps to minimize the interference from external noise. Considering the typical operating environments of AC contactors, this paper, in reference to the Chinese National Standard GB 14048.4-2010, organizes the experimental conditions of the platform into a tabular form, as shown in Table 1.

**Table 1. Experimental conditions**

| Parameter | Value | Parameter | Value |
| --- | --- | --- | --- |
| Coil voltage/V | AC 220 | Work system | AC-4 |
| Load voltage/V | 400(380) | Load current/A | 222 |
| Load type | RL | Power factor | 0.35 |
| Operating frequency/(times/h) | 300 | Sampling frequency/Hz | 1M |

**Comment 9:**The paper introduces a new feature selection method (BO-SGRRF) but fails to adequately justify its superiority over existing methods. There is a lack of comparative analysis with other feature selection techniques, making it unclear why this particular method was chosen.

**Response:**

Thank you for your valuable feedback! In the introduction of our latest manuscript, we've included a straightforward comparison of existing feature selection methods, highlighting their strengths and weaknesses, and have introduced the approach our paper takes. Further into the manuscript, specifically on pages 14 to 15, we provide a detailed comparison between our method and others, clearly demonstrating that our approach is superior. We appreciate your insights as they help us improve the clarity and impact of our work.

......Statistical analysis of the time-domain waveform and frequency-domain waveform of vibration signals can yield high-dimensional feature data, such as amplitude, amplitude standard deviation, and amplitude variance. It is known from statistical knowledge that the standard deviation is closely related to variance, implying potential redundancy in high-dimensional features where multiple features may be linearly correlated, thus leading to reduced accuracy or efficiency of the state recognition model. Mainstream feature selection methods include the correlation coefficient method, embedded method, filter method, and recursive elimination method. The correlation coefficient method is relatively straightforward, but its threshold setting is subjective, requiring multiple judgments to select optimal features, resulting in low efficiency for high-dimensional datasets [14-18]. The embedded method is suitable for high-dimensional datasets, often using the random forest model as the base learner. Compared to the correlation coefficient method, the embedded method significantly improves feature selection efficiency. However, due to its unbiased nature, the embedded method may split nodes along directions of non-critical features, affecting the model's decision-making process [19-27]. The recursive elimination method exhaustively searches all possible feature combinations, which is beneficial for identifying true key features, but it is time-consuming on high-dimensional datasets and dependent on the type of base learner. Therefore, this paper introduces a regularization term into the random forest, biasing it and reducing consideration of unimportant features. Concurrently, this paper integrates the improved random forest with the concept of recursive elimination, addressing the dependency of the recursive elimination method on the base learner type, and combines different features based on feature importance, solving the time-consuming issue of enumeration and efficiently selecting key features......

......It is evident from Table 3 that the embedding method fails to effectively select key features. This is attributed to the RF's unbiased node splitting direction, designed to accommodate various tasks, which results in an overemphasis on minor gain directions and an inability to utilize known information to guide the splitting direction. In contrast, RFRS and the other two feature selection methods have efficiently reduced the feature scale, and they collectively identify T1 (time-domain peak-to-peak amplitude), T5 (time-domain amplitude standard deviation), and T7 (time-domain skewness factor) as the most critical vibration features. However, it should be noted that there are distinct differences in the decision-making for other key features among these three feature selection methods: RFRS considers T3, T6, and F6 as key features; Spearman's rho regards T6 and F6 as key features; and the Filter method identifies F2 and F10 as key features. To evaluate the merits of these three feature selection methods, this study assesses the features using four common classifiers, with the metrics of recall rate, F1-Score, and precision. The corresponding results are depicted in Table 4.

**Table 3　Results of feature selection**

| Feature selection method | Feature set | Selection result |
| --- | --- | --- |
| RFRS | T1-T7  F1-F10 | T1,T3,T5,T6,T7,F6 |
| Spearman's Rho |  | T1,T5,T6,T7,F6 |
| Embedded |  | T1-T7  F1-F10 |
| Filter |  | T1,T5,T7,F2,F10 |

**Table 4　Performance of different feature selection methods on classifiers**

| Feature selection method | Classifier | Recall | F1-Score | Precision |
| --- | --- | --- | --- | --- |
| RFRS | LGBM | 90.78% | 90.72% | 91.70% |
|  | KNN | 87.51% | 87.56% | 88.65% |
|  | SVM | 87.49% | 87.57% | 88.47% |
|  | LR | 83.69% | 83.78% | 84.71% |
| Spearman's Rho | LGBM | 90.18% | 90.14% | 91.09% |
|  | KNN | 86.67% | 86.73% | 87.56% |
|  | SVM | 87.27% | 87.33% | 88.17% |
|  | LR | 84.12% | 84.20% | 85.40% |
| Embedded | LGBM | 88.69% | 88.66% | 89.92% |
|  | KNN | 83.78% | 83.91% | 84.88% |
|  | SVM | 87.07% | 87.15% | 87.82% |
|  | LR | 83.77% | 83.83% | 84.48% |
| Feature selection method | Classifier | Recall | F1-Score | Precision |
| Filter | LGBM | 87.29% | 87.35% | 87.98% |
|  | KNN | 85.11% | 85.19% | 85.82% |
|  | SVM | 86.72% | 86.76% | 87.27% |
|  | LR | 82.95% | 83.0% | 83.86% |

The results from the classifiers reveal that the Embedded method, despite incorporating all features as inputs to the model, yields lower scores across the three metrics compared to the other three feature selection methods. This observation suggests that there is not always a positive correlation between the number of features and model performance; in fact, an increased number of features can often lead to a decrease in model performance. Consequently, the selection of features prior to data analysis or decision-making is a crucial measure to ensure the efficacy of the research.

As depicted in Table 4, the average scores for Recall, F1-Score, and Precision for RFRS are 87.37%, 87.41%, and 84.71%, respectively; for Spearman's rho, the corresponding scores are 82.06%, 87.1%, and 88.06%, respectively; and for the Filter method, the scores are 85.52%, 85.58%, and 86.23%, respectively. Comparatively, the Recall score of RFRS is 5.31% higher than that of Spearman's rho and 1.85% higher than the Filter method; its F1-Score is 0.31% higher than Spearman's rho and 1.83% higher than the Filter method; whereas its Precision score is 3.35% lower than Spearman's rho and 1.52% lower than the Filter method. These outcomes indicate that RFRS surpasses Spearman's rho, the Filter method, and the Embedded method in the state identification of stages II and III of AC contactors. Conversely, in the state identification of stage I, RFRS is less effective than Spearman's rho and the Filter method, as it may erroneously classify stage I samples as belonging to stages II and III, which could result in premature alerts in practical scenarios. However, when considering the F1-Score—a comprehensive metric that encapsulates both Recall and Precision—RFRS is favored over the other feature selection methodologies......

**Comment 10:**The statistical analysis presented in the paper, particularly regarding the optimization process and feature selection, appears to lack rigor. The choice of optimization algorithms seems arbitrary, and there is insufficient explanation of how parameter values were selected.

**Response:**

Thank you so much for your valuable feedback!Based on your third and fifth suggestions, we have realized that Bayesian optimization did not enhance the model's performance as expected, but rather complicated the issue. Additionally, considering the self-optimizing capabilities of the random forest model, we have decided to remove the content regarding Bayesian optimization. It should be noted that we have selected features commonly used in the fault diagnosis studies of circuit breakers, motors, and other equipment, which has led to an increase in the dimensionality of the feature set. The purpose of this study is precisely to provide key features for future research on AC contactors.

**Comment 11:**The interpretation of results, especially regarding the significance of selected features and their correlation with contactor degradation states, is vague and lacks clarity. The paper fails to provide a clear link between the extracted features and the observed vibration patterns.

**Response:**

Thank you sincerely for your feedback! We apologize for not providing a clear enough explanation of our experimental results earlier. In our latest manuscript, we have made a comprehensive update to all our findings, covering aspects such as data processing, label creation, and the relationship between vibration features and the states of the contactors. We appreciate your patience and guidance as we strive to improve our work.

Due to space limitations, we have only included a selection of the content here for your reference:

When samples are entered into the offline database, accurate classification of the samples is crucial for subsequent data analysis or decision-making activities. This paper, taking the CJX2-5011 model AC contactor as an example, elaborates on the criteria for sample classification.

In Section 2.1, the working principle and failure mechanisms of AC contactors are described in detail. Research indicates that the failures of AC contactors are primarily divided into two types: welding and mechanical interlocking. Although these two failure phenomena have different manifestations, they are consistent in physical essence (there is a deviation in the relative position of the moving and static contacts). As shown in Fig 3, the kinematic manifestation of the welding phenomenon is that after the moving and static contacts are engaged, the moving contact continues to move forward for a certain distance, a process that can be characterized by overtravel. The kinematic manifestation of the mechanical interlocking phenomenon is that after the moving and static contacts are engaged, they cannot separate normally, a process that can be characterized by the minimum distance between the moving and static contacts (opening distance). By measuring the relative position between the moving and static contacts, the current working state of the contacts can be determined.

Fig 5 demonstrates the trends in overtravel and opening distance variation across the entire operational lifespan of the contactor. The change in opening distance shows a periodic characteristic, evident in three distinct stages: Stage I (with the number of operations ranging from 10,000 to 30,000 times), Stage II (with the number of operations ranging from 30,000 to 70,000 times), and Stage III (with the number of operations ranging from 70,000 to 90,000 times), where peak phenomena are

**Fig.5 Trends in overtravel and opening distance variations**

observed in each stage. Compared to the opening distance, the variation in overtravel exhibits a stepwise pattern, aligning well with the irreversible nature of contact aging. During Stage I (with the number of operations ranging from 10,000 to 40,000 times), the amplitude change of overtravel is not pronounced; after Stage II (with the number of operations ranging from 40,000 to 70,000 times), there is a marked increase in the amplitude of overtravel; and in Stage III (with the number of operations ranging from 70,000 to 90,000 times), the rate of increase in the amplitude of overtravel accelerates. Based on the above analysis, this study selects the change in overtravel as the primary criterion for classifying samples.

**Comment 12:**Figures and tables are poorly presented and difficult to interpret. The lack of clear labeling and inconsistent formatting detracts from the readability of the paper and hinders understanding of the experimental process and results.

**Response:**

Thank you for your guidance! We have now standardized the presentation of our charts and sincerely apologize for any inconsistencies previously.

**Comment 13:**The excessive use of acronyms and technical jargon throughout the paper makes it inaccessible to readers who may not be intimately familiar with the specific terminology of the field. This limits the paper's audience and detracts from its overall impact.

**Response:**

Thank you for your careful guidance! We have rewritten the paper and reduced the use of technical jargon to ensure that it is more accessible and understandable for our readers.

**Comment 14:**The experimental setup and conditions described in the paper are specific to a particular type of contactor under certain operating conditions. As a result, the findings may lack generalizability to other contactor systems or real-world applications, limiting the broader relevance of the research.

**Response:**

Thank you for your meticulous guidance! We would like to clarify that the AC contactors used in our experiments are the most widely used type on the market. Additionally, the aging mechanism of all contactors is generally the same, primarily caused by the erosive action of electrical arcing. Therefore, our test subjects are highly representative. Meanwhile, to ensure that our research findings can be effectively applied in the real world, our experimental conditions were set according to Chinese national standards, which every contactor must meet during actual operation.

**Comment 15:**The paper's conclusion is relatively weak and fails to clearly articulate the implications of the research findings. There is a lack of discussion on how the results could be applied in practical settings or how they contribute to advancing the understanding of contactor behavior.

**Response:**

Thank you for your feedback! We understand your concerns. In the latest manuscript, we have disclosed additional experimental details and data processing procedures, and we have revised the conclusions of the paper to ensure that readers can fully understand our contributions.

**5. Conclusion**

This study proposes a feature selection method based on Regularized Random Forest with Recursive Selection (RFRS) to address the issue of feature redundancy in the vibration signals of AC contactors. Through comprehensive analysis and experimental validation, the following conclusions have been drawn:

(1)The RFRS feature selection method introduced in this study effectively extracts key vibration features of AC contactors and reduces the dimensionality of the feature set, outperforming current mainstream feature selection methods.

(2)In the identification of the aging state of AC contactors (stages II and III), the performance of RFRS is 5.31% and 1.85% higher than that of Spearman's rho and the Filter method, respectively. Additionally, this study found that RFRS is more prone to classifying the normal state (stage I) as an aging state (stages II and III) compared to the two aforementioned methods. After considering these two characteristics comprehensively, the performance of RFRS is 0.31% and 1.83% higher than that of Spearman's rho and the Filter method, respectively. These results suggest that RFRS is generally superior to current mainstream feature selection methods, although the specific application scenarios still require researchers to make decisions based on actual needs.

(3)There remain numerous challenges in conducting state identification research on AC contactors from the perspective of vibration signals, particularly in how to extract features with more pronounced trends or more stable changes from the vibration signals. Therefore, future work will focus on two aspects: one is the continuous expansion of the database; the other is the improvement of data quality and the exploration of new feature extraction techniques. In summary, the RFRS-based feature selection method proposed in this study has demonstrated its effectiveness in resolving the issue of time-frequency domain feature redundancy in the vibration signals of AC contactors, compared to traditional methods.

In summary, the feature selection method based on Regularized Random Forest with Recursive Selection (RFRS) proposed in this study has demonstrated its effectiveness in addressing the issue of time-frequency domain feature redundancy in the vibration signals of AC contactors, showing superiority over traditional methods.

**Comment 16:**Overall, the paper suffers from poor writing quality, including grammatical errors, awkward phrasing, and disjointed structure. These issues detract from the professionalism of the paper and may undermine the credibility of the research.

**Response:**

Thank you for your valuable feedback! We sincerely apologize for the previous quality of our manuscript. Under your guidance, we have thoroughly rewritten the paper and earnestly request that you continue to provide us with your insightful suggestions!
