# Supplementary material for: Research on feature selection for AC contactor vibration signals based on regularized random forest with recursive selection
Source: PLoS One. 2024 Sep 6;19(9):e0310110. doi: 10.1371/journal.pone.0310110 (PMC11379156; doi:10.1371/journal.pone.0310110)
Supplement: Attachment — Submitted filename: Response to reviewers.docx [file pone.0310110.s002.docx]

Dear Editor and Reviewers,

On behalf of all the authors, I would like to express our sincere gratitude for the thorough review and valuable feedback provided on our manuscript (Manuscript ID: PONE-D-24-16516, Title: "Research on Feature Selection for AC Contactor Vibration Signals Based on Regularized Random Forest with Recursive Selection"). We are honored by your affirmation and suggestions, which have been instrumental in enhancing the quality of our manuscript and refining the details of our conclusions.

We have taken great care to address each of your points and have made comprehensive revisions to the manuscript. The main revisions include:

**1.Adjustment of Main Text:** We have refined the abstract, introduction, and conclusion, corrected any inappropriate descriptions, and more prominently highlighted the contributions of our study.

**2.Improvement of Language Standards:** We have made precise adjustments to the definitions of acronyms, the consistency of titles, and the use of technical terms to ensure the accuracy and professionalism of our expressions..

**3.Supplement of Experimental Details:** We have added detailed information regarding the duration of the vibration signals, sampling frequency, and the division of the dataset to enhance the transparency and reproducibility of the paper.

**4.Optimization of Figures and Tables:** We have redesigned the figures to ensure that all text and information within them are clearly visible and have used tables to provide detailed descriptions of multiple features, thereby improving the readability of the paper.

**5.Completion of Model-Related Settings:** We have added descriptions of comparative methods, classifiers, and evaluation metrics to provide a clearer understanding of why we chose these methods. Additionally, we have included results from both the training and testing datasets to ensure the reliability of our conclusions.

To clearly demonstrate our consideration and response to each of your comments, we have implemented a color-coding system in the revised manuscript. Direct responses to your feedback are highlighted in red, while modifications and additions are indicated in blue. We hope this approach will facilitate the identification of our improvements and assist in an efficient re-review process.

We eagerly await further feedback and are committed to continuing to enhance the quality of the manuscript. We kindly ask you to review the revised version and provide any additional suggestions or queries. It is a privilege to collaborate with you in advancing the frontiers of scientific research.

We appreciate your consideration and support once again.

Sincerely,

The Authors

**Response to Reviewer 1**

The paper introduces an approach for feature selection using a regularized random forest and a recursive selection procedure, but with the traditional regularization term being modified to include the ratio of importance, relative to the maximum importance among all features, of a certain feature in the penalization. A total of 7 time-domain and 10 frequency-domain features are analyzed and selected, via both traditional feature selection methods and the proposed one, and then evaluated using four classifiers. The paper’s results show some advantage of the proposed method over other traditional feature selection methods. Some comments:
We extend our heartfelt thanks for your affirmation and support! Your well-considered suggestions have drawn our attention to details that were previously overlooked. By meticulously addressing these aspects, we have significantly enhanced the quality of our manuscript.

**Note:** In this revision, we have uploaded the following documents: Cover Letter, Figure, Response to Reviewers, Manuscript, and Revised Article with Changes Highlighted.

**1.Response to Reviewers:** We have adopted a dual-color style, with red font indicating our responses to the reviewers' suggestions, and blue font representing the content modified according to the suggestions.

**2.Revised Article with Changes Highlighted:** To facilitate effective re-review by the reviewers, this document retains the original format, with figures directly inserted into the text.

**3.Manuscript:** To comply with publication standards, this document has been adjusted according to the PLOS ONE style. Figures have been processed with the PACE tool and uploaded as separate files.

**Comment 1:**Review of acronyms’ definitions along all paper. Many acronyms are defined multiple times, which is not correct. For example, the “(RFRS)” acronym definition appears in the abstract, in the keywords, in the last paragraph of the introduction and then twice in the Conclusion section. Other acronyms are also defined multiple times, such as “(RF)”. Please revise those acronyms over the entire paper and only define them the first time they appear.
**Response:**

We greatly appreciate your valuable suggestions, which have prompted us to re-examine the standardization of our manuscript. We realized that, although our intention was to accommodate readers unfamiliar with specialized terminology, the over-definition of acronyms actually deviated from the norms of academic writing. To rectify this, we have meticulously adjusted the abstract, keywords, introduction, and conclusion of our manuscript, ensuring that each acronym is defined only upon its first occurrence. This approach maintains both the clarity of the text and the rigor of academic writing.

The revised content is located on page 1 (Abstract and Keywords), pages 1-3 (Introduction), and pages 15-16 (Conclusion) of the 'Revised Article with Changes Highlighted'. (Or, see page 2 (Abstract), pages 2-4 (Introduction), and pages 18-19 (Conclusion) in the 'Manuscript'.) The following displays the modified sections in 'Revised Article with Changes Highlighted':

**Abstract:** When conducting condition recognition research on AC contactor vibration signals through time-frequency analysis, the feature data exhibit a high degree of redundancy, which leads to repetitive information and hinders the accuracy of recognition. To address the redundancy issue in the features of AC contactor vibration signals, this study introduces a feature selection method based on Regularized Random Forest with Recursive Selection (RFRS). Initially, a test platform for AC contactor vibration signals was established, and time-frequency domain features of the AC contactor vibration signals were extracted. Subsequently, the traditional Random Forest (RF) was refined by optimizing its stopping criteria using the Recursive Feature Elimination approach and by incorporating a regularization coefficient during the splitting process to direct the split towards significant features. This modification not only enhances the Random Forest's capacity to leverage existing information but also introduces a bias, enabling it to favor important features. Finally, through case analysis, the proposed method effectively reduced the dimensionality of the feature set and achieved an average of 87.37% for Recall, 87.41% for F1-Score, 88.38% for Precision, and 85.74% for Accuracy. The overall performance of this method surpasses that of the three mainstream feature selection methods: Spearman's rank correlation coefficient method, the embedded method, and the filter method. This study thus provides a rather effective feature selection approach for the state recognition study of AC contactors.

**Keywords:** Vibration signal, Feature selection, Regularized random forest with recursive selection, AC contactors, Condition identification, Time-Frequency domain features, Machine learning

**Introduction: ......**In summary, to further explore the application of vibration signals in the field of state recognition for AC contactors and to address the issue of high redundancy in both time-domain and frequency-domain features of these signals, this study proposes a vibration feature selection method for AC contactors based on RFRS. Firstly, this study constructs an experimental platform for AC contactors based on vibration signals, in accordance with relevant testing standards. Subsequently, the platform is utilized to extract both time-domain and frequency-domain features of the vibration signals. Furthermore, by introducing a regularization term and integrating the concept of recursive feature elimination, improvements are made to the random forest algorithm, enabling it to effectively address the selection of time-frequency domain features from AC contactor vibration signals.

# **Conclusion:** This study proposes a feature selection method based on RFRS to address the issue of feature redundancy in the vibration signals of AC contactors. Through comprehensive analysis and experimental validation, the following conclusions have been drawn**......**

# ****Comment 2:****3.3.1 Feature Extraction: pages 8-9 include the name, description and formula of multiple features, organized per paragraph. This information would be better suited in a table with three columns: name, description and formula.

# ****Response:**** We sincerely appreciate your valuable guidance. Although we hesitated during the manuscript preparation whether to present the feature extraction information in tabular form, concerned it might not adhere to academic standards, your suggestions have illuminated the path forward. Accordingly, we have revised the manuscript in accordance with your feedback, presenting the names, descriptions, and formulas of features in a three-column table in Section 3.3.1 as Table 2. This change not only enhances the readability of the manuscript but also renders our report more concise and clear, aligning with the best practices in academia.

# We express our heartfelt thanks once again for your meticulous review and constructive comments, which have been pivotal in elevating the quality of our paper.

The revised content is located in section 3.3.1, Table 2, on pages 7-8 of the 'Revised Article with Changes Highlighted'. (Or, see section 2.3.1, Table 2, on pages 9-10 in the 'Manuscript'.) The following displays the modified sections in 'Revised Article with Changes Highlighted':

**Table 2. List of time-frequency domain feature**

| Name | Formula | Description |
| --- | --- | --- |
| Time-domain peak-to-peak amplitude (T1) | $\text{T}1=\max(x_{i})-\min(x_{i})$ | The difference between the maximum and minimum signal values within a specified time frame, indicating the signal's range of variation. |
| Time-domain amplitude mean (T2) | $\text{T}2=(\sum_{i=1}^{N} x_{i})/N$ | The average value of the signal amplitudes over a specified time interval, reflecting the central tendency of the signal's amplitude. |
| Time-domain amplitude absolute mean (T3) | $\text{T}3=(\sum_{i=1}^{N} \left\vert x_{i}\right\vert)/N$ | The mean value of the absolute signal amplitudes over a specified time period, indicating the average energy of the signal. |
| Time-domain amplitude root mean square (T4) | $\text{T4=}\sqrt{(\sum_{i=1}^{N} x_{i}^{2})/N}$ | The square root of the mean of the squares of the signal amplitudes over a specified time period, a measure of the effective value of the signal's amplitude. |
| Time-domain amplitude standard deviation (T5) | $\text{T5=}\sqrt{(\sum_{i=1}^{N} (x_{i}-\text{T2})^{2})/N}$ | A measure of the amount of variation or dispersion of a set of signal amplitudes from their mean value. |
| Kurtosis factor (T6) | $\text{T6}=(\text{T2}/\text{T5})^{4}$ | A statistical measure that describes the tail weight of the amplitude distribution in comparison to a normal distribution, indicating the peakedness or flatness of the signal distribution. |
| Skewness factor (T7) | $\text{T7}=(\text{T2}/\text{T5})^{3}$ | A statistical measure that quantifies the asymmetry of the amplitude distribution around its mean, indicating the direction and degree of the data's departure from normal distribution symmetry. |
| Waveform factor (F1) | $\text{F1}=\text{T4}/\text{T3}$ | The ratio of the root mean square value to the mean value of the signal amplitude, reflecting the waveform's fluctuation characteristics. |
| Peak factor (F2) | $\text{F2}=\text{T1}/\text{T4}$ | The ratio of the peak amplitude to the root mean square value of the signal, indicating the relative magnitude of the highest peak in comparison to the average energy of the signal. |
| Impulse factor (F3) | $\text{F3}=(\text{T1}/\text{T3})^{3}$ | A measure of the impulsive characteristics of the signal, typically indicating the presence of short-duration, high-amplitude events. |
| Safety margin factor (F4) | $\text{F4}=\text{T1}/(\text{T3)}^{2}$ | A measure of the safety margin or the difference between the system's stability threshold and its current operating point, reflecting the system's resilience to disturbances. |
| Frequency-domain peak-to-peak amplitude (F5) | $\text{F5}=\max(X_{i})-\min(X_{i})$ | The difference between the maximum and minimum amplitude values in the frequency domain, indicating the range of amplitude variation across frequencies. |
| Frequency-domain amplitude mean (F6) | $\text{F6}=(\sum_{i=1}^{N} X_{i})/N$ | The average value of the amplitudes across the frequency spectrum, providing a measure of the central tendency of the signal's energy distribution. |
| Frequency-domain amplitude median (F7) | $\text{F7}=\text{median}(X_{i})$ | The median value of the spectral amplitudes when sorted in ascending order, used to mitigate the impact of extreme values on the analysis. |
| Frequency center (F8) | $\text{F8}=(\sum_{i=1}^{N} f_{i}\times X_{i})/(\sum_{i=1}^{N} X_{i})$ | The average value of the upper and lower cutoff frequencies, representing the central frequency of the signal's amplitude distribution in the frequency domain. |
| Frequency root mean square (F9) | $\text{F9}=(\sum_{i=1}^{N} f_{i}^{2}\times X_{i})/(\sum_{i=1}^{N} X_{i})$ | The square root of the mean of the squares of the amplitudes across the frequency spectrum, indicating the effective amplitude level of the signal in the frequency domain. |
| Frequency standard deviation (F10) | $\text{F10}=\sqrt{\sum_{i=1}^{N} (f_{i}-\text{F8})^{2}\times X_{i}/\sum_{i=1}^{N} X_{i}}$ | A measure of the dispersion or spread of the amplitudes across the frequency spectrum, indicating the variability of the signal's energy distribution. |

In Table 2, *x_i_* denotes the amplitude of the *i*-th sample in the time domain, *X_i_* represents the amplitude at the *i*-th frequency point, *f_i_* refers to the frequency-domain value.

# ****Comment 3:****Sections 3.3.1 and 3.3.2 have the same name “Feature Extraction”. I also believe section 3.3.2 is related to experiment and/or some sort of data processing, not Feature Extraction itself, so this name does not seem not suitable unless extra information is provided in the section’s title. ****Response:****

# We sincerely appreciate the careful examination and valuable feedback you have provided. The issue you identified with the duplicate titles for Sections 3.3.1 and 3.3.2 has indeed highlighted an error in our manuscript. We offer our sincere apologies for this mistake and any confusion it may have caused.

# Understanding the importance of clear and distinct section titles for the paper's readability and scholarly quality, we have made the following amendments to the manuscript:

# Section 3.3.1, which concentrates on extracting time-frequency domain features from vibration signals, retains the title "Feature extraction."

# Section 3.3.2, dealing with the sample classification approach and distinct from the feature extraction process, has been correctly retitled "Sample classification."

# These revisions have improved the titles' clarity and descriptiveness, making the research process more understandable and coherent. We trust that these changes will help readers more easily follow our research and strengthen the paper's overall standard.

# Thank you once again for your professional review and insightful advice. Your comments are extremely valuable in assisting us to refine the paper's content and enhance its academic precision.

The revised content is located in section 3.3.1 on page 7 and section 3.3.2 on page 8 of the 'Revised Article with Changes Highlighted'. (Or, see section 2.3.1 on page 9 and section 2.3.2 on page 10 in the 'Manuscript'). The following displays the modified sections in the 'Revised Article with Changes Highlighted':

**3.3.1 Feature extraction**

As can be inferred from the fault principles of AC contactors in Section 3.1, welding or mechanical interlocking phenomena can lead to uncertainties in the separation action**......**

**3.3.2 Sample classification**

When samples are entered into the offline database, accurate classification of the samples is crucial for subsequent data analysis or decision-making activities......

# ****Comment 4:****Section 3.3.2 Feature Extraction: in the last paragraph of page 9, Stage I is stated as # of operations ranging from 10000 to 30000 and Stage II from 30000 to 70000. In the first paragraph of page 10, Stages I and II are stated as from 10000 to 40000 and 40000 to 70000 instead. Which one is the correct value? The first time (page 9) the stages are cited must be adjusted to the correct value, while the second time (page 10) should not include the parenthesis part after ‘Stage X’ as the range for each stage was already described in the previous paragraph. ****Response:****

# We sincerely appreciate your meticulous guidance and valuable feedback. In Section 3.3.2, we introduced a sample classification method based on two key physical quantities—overtravel and opening distance—that characterize the relative position between the moving and static contacts of the AC contactor. Each of these quantities exhibits distinct trends. When classifying states based on opening distance, Stage I is defined as the range from 1 to 30,000; whereas, using overtravel as the classification criterion, Stage I extends from 1 to 40,000. After thorough analysis and considering the change characteristics of these two physical quantities as well as the irreversible nature of contactor degradation, we ultimately determined that overtravel should be the primary basis for state classification.

# However, your comments have made us realize that an analysis without emphasis and the repetitive use of terminology might confuse readers. Therefore, we have made adjustments to the relevant parts of the manuscript, focusing on a detailed analysis of overtravel and standardizing and optimizing all descriptions related to stage classification to ensure the section's conciseness and clarity.

# We believe these adjustments not only address the issues you raised but also make our research methods and results more understandable.

The revised content is located in section 3.3.2 on pages 8-9 of the 'Revised Article with Changes Highlighted'. (Or, see section 2.3.2 on pages 10-11 in the 'Manuscript'). The following displays the modified sections in the 'Revised Article with Changes Highlighted':

# **3.3.2 Sample classification**

**......**

# As depicted in Fig 5, this study presents the variations in overtravel and opening distance of the contactor throughout its entire service life. The change in opening distance exhibits a certain periodic characteristic. In contrast, the variation in overtravel demonstrates a stepwise pattern, aligning with the irreversible degradation of the contact surface. During Stage I (with the number of operations ranging from 1 to 40,000 times), the amplitude change of overtravel is not significant; after Stage II (with the number of operations ranging from 40,000 to 70,000 times), there is a noticeable increase in the amplitude of overtravel; and in Stage III (with the number of operations ranging from 70,000 to 90,000 times), the rate of increase in overtravel amplitude accelerates. Therefore, based on the three stages of overtravel change, samples can be categorized into three states: normal, mildly degraded, and severely degraded.

**Fig.5 Trends in overtravel and opening distance variations**

# ****Comment 5:****Fig 8: I believe this graph is relative to the three Stages described in section 3.3.2, but its legend uses the term ‘Phase X’ instead, mentioned nowhere in the paper. Either describe the term along the paper, if it is supposed to be Phase, or change it to ‘Stage X’ instead.

# ****Response:****

# We sincerely appreciate your meticulous review and valuable feedback. The issue you highlighted that the legend should use “Stage X” instead of “Phase X” is indeed a discrepancy that we overlooked, for which we sincerely apologize.

# Following your guidance, we have made the necessary corrections to the legend, replacing the inappropriate term "Phase X" with the consistent "Stage X" used throughout the paper. This amendment ensures the uniformity and accuracy of the terminology, aiding readers in better correlating the graphical content with the textual descriptions.

# We are committed to meticulously attending to every detail of the paper to uphold academic rigor. Your professional critique and constructive suggestions are greatly valued once again.

The revised content is located at Fig 9 on page 11 of the 'Revised Article with Changes Highlighted'. (Or, see Fig 9 on page 13 in the 'Manuscript'). The following displays the modified sections in the 'Revised Article with Changes Highlighted':

**Fig.9 Recognition accuracy for three stages across different feature combinations**

****Comment 6:****Table 4 includes the metrics Recall, F1-Score and Precision for the four feature selection methods analyzed and for four different classifiers. Accuracy should also be included in the table. Although generic and misleading in some cases, it is still an easy-to-interpret metric that, alongside the others already provided by the authors, helps understanding the advantages of the proposed approach.

# ****Response:****

We sincerely appreciate your valuable suggestions. We recognize that, while the Accuracy metric may be misleading in some situations, it is also a universally understood and easily interpretable measurement standard. Compared to metrics such as Recall, F1-Score, and Precision, Accuracy offers a broad perspective that helps readers quickly grasp the advantages of the methods we propose.

In light of this, we have included the Accuracy metric in the latest revision of our manuscript to provide a more comprehensive assessment of the performance of the RFRS method and various feature selection methods (including Spearman's rank correlation method, embedded method, and Filter method). We believe that this addition not only enriches our analysis but also provides readers with an additional intuitive evaluation tool.

We are grateful for your feedback and are confident that these enhancements will make our research more rigorous and convincing.

The revised content is located in section 4.3, Tables 4 and 5, on pages 13-15 of the 'Revised Article with Changes Highlighted'. (Or, see section 3.3, Tables 4 and 5, on pages 15-18 in the 'Manuscript'). The following displays the modified sections in the 'Revised Article with Changes Highlighted':

**Table 4　Performance metrics on the training dataset**

| Method | Classifier | Recall | F1-Score | Precision | Accuracy |
| --- | --- | --- | --- | --- | --- |
| RFRS | LGBM | 94.44% | 94.44% | 94.46% | 94.44% |
|  | KNN | 92.31% | 92.29% | 92.30% | 92.29% |
|  | SVM | 89.19% | 89.14% | 89.13% | 89.14% |
|  | LR | 86.08% | 86.07% | 86.08% | 86.07% |
|  | Average Value | 90.51% | 90.49% | 90.49% | 90.49% |
| Spearman's Rho | LGBM | 93.58% | 93.58% | 93.60% | 93.58% |
|  | KNN | 91.28% | 91.26% | 91.27% | 91.28% |
|  | SVM | 88.61% | 88.57% | 88.58% | 88.61% |
|  | LR | 86.57% | 86.55% | 86.56% | 86.57% |
|  | Average Value | 90.01% | 89.99% | 90.0% | 90.01% |
| Embedded | LGBM | 95.40% | 95.40% | 95.42% | 95.40% |
|  | KNN | 92.14% | 92.14% | 92.16% | 92.14% |
|  | SVM | 90.60% | 90.60% | 90.61% | 90.60% |
|  | LR | 86.70% | 86.67% | 86.68% | 86.67% |
|  | Average Value | 91.21% | 91.20% | 91.22% | 91.20% |
| Filter | LGBM | 91.84% | 91.81% | 91.84% | 91.84% |
|  | KNN | 91.50% | 85.19% | 91.51% | 91.50% |
|  | SVM | 89.44% | 89.40% | 89.41% | 89.44% |
|  | LR | 85.74% | 85.69% | 85.67% | 85.74% |
|  | Average Value | 89.63% | 88.02% | 89.61% | 89.63% |

**Table 5**　**Performance metrics on the test dataset**

| Method | Classifier | Recall | F1-Score | Precision | Accuracy |
| --- | --- | --- | --- | --- | --- |
| RFRS | LGBM | 90.78% | 90.72% | 91.70% | 88.52% |
|  | KNN | 87.51% | 87.56% | 88.65% | 85.27% |
|  | SVM | 87.49% | 87.57% | 88.47% | 86.45% |
|  | LR | 83.69% | 83.78% | 84.71% | 82.73% |
|  | Average Value | 87.37% | 87.41% | 88.38% | 85.74% |
| Spearman's Rho | LGBM | 90.18% | 90.14% | 91.09% | 88.19% |
|  | KNN | 86.67% | 86.73% | 87.56% | 85.15% |
|  | SVM | 87.27% | 87.33% | 88.17% | 85.95% |
|  | LR | 84.12% | 84.20% | 85.40% | 83.33% |
|  | Average Value | 87.06% | 87.10% | 88.06% | 85.66% |
| Embedded | LGBM | 88.69% | 88.66% | 89.92% | 88.69% |
|  | KNN | 83.78% | 83.91% | 84.88% | 83.78% |
|  | SVM | 87.07% | 87.15% | 87.82% | 87.07% |
|  | LR | 83.77% | 83.83% | 84.48% | 83.77% |
|  | Average Value | 85.83% | 85.89% | 86.78% | 85.83% |
| Filter | LGBM | 87.29% | 87.35% | 87.98% | 87.29% |
|  | KNN | 85.11% | 85.19% | 85.82% | 85.11% |
|  | SVM | 86.72% | 86.76% | 87.27% | 86.72% |
|  | LR | 82.95% | 83.0% | 83.86% | 82.96% |
|  | Average Value | 85.52% | 85.58% | 86.23% | 85.52% |

# Table 4 presents the performance of various classifiers on the training dataset. When considering the average scores across the four metrics, the embedded method demonstrates superior learning capability for known samples compared to other approaches. However, this method fails to effectively select key features, instead incorporating all features into the learning process. On the premise of effectively reducing feature dimensionality, RFRS outperforms Spearman's Rho and the Filter in terms of learning ability for known samples.

# Table 5 illustrates the performance of various classifiers on the test dataset. An integrated analysis of Tables 4 and 5 reveals that the embedded method, hindered by feature redundancy, exhibits inferior generalizability and lower recognition capability for unknown samples compared to RFRS and Spearman's Rho. Furthermore, Table 5 demonstrates that RFRS outperforms both Spearman's Rho and the Filter across all four evaluation metrics. Specifically, in terms of Recall, RFRS achieves an average of 87.37%, slightly higher than Spearman's Rho at 87.06%, and significantly better than the Filter, which stands at 85.52%. In Precision, RFRS shows an average of 88.38%, surpassing the 88.06% of Spearman's Rho and the 86.23% of the Filter, indicating its high efficiency in reducing false positives. The F1-Score reflects RFRS's balanced capability when considering both Recall and Precision, with an average F1-Score of 87.41%, outperforming the 87.10% of Spearman's Rho and the 85.58% of the Filter. Although RFRS has a slightly lower average accuracy at 85.74% compared to Spearman's Rho at 85.66%, it still exceeds the Filter's 85.52%, with the differences being minimal, indicating that RFRS is not inferior in overall recognition performance. These results highlight the significant advantages of RFRS in the selection of time-frequency domain features for the vibration signals of AC contactors, providing an effective feature selection tool for condition recognition research.

# ****Comment 7:****4.3 Results and Analysis: some more robust classifiers could be evaluated, such as MLP and a traditional Random Forest classifier. ****Response:****

We extend our heartfelt thanks for your valuable comments. The aim of our study is to provide an effective feature selection method for the state recognition research of AC contactors, with the goal of reducing feature dimensions and enhancing recognition accuracy. In the evaluation process, we selected classifiers from different learning paradigms, including the Light Gradient Boosting Machine (LGBM), a tree-based model that outperforms traditional random forests in terms of computational efficiency and performance.

In response to your suggestions regarding the MLP and traditional random forest classifiers, we have considered the following points:

**1.Overfitting Risk:** Deep learning models such as MLP excel with large datasets but are at a higher risk of overfitting with smaller datasets. Given the limited size of the AC contactor dataset, we opted for classifiers more suitable for small-sample data, such as Support Vector Machine (SVM) and K-Nearest Neighbors (KNN), to objectively assess the impact of feature selection methods on classification performance.

**2.Practical Requirements:** Although deep learning models may surpass traditional classifiers in performance, the core of our research lies in feature selection. We wish for the RFRS method to be adaptable to classifiers of various learning paradigms, including distance-based classifiers and ensemble learning classifiers, thereby offering a more robust feature selection strategy for state recognition research.

Additionally, the RFRS method we propose and the comparative method based on the embedded approach are both improvements upon the traditional random forest. Therefore, we did not use the traditional random forest as an evaluation classifier in this study.

We believe that these considerations and choices ensure that our research is innovative and meets the needs of practical applications. We look forward to your further feedback and are committed to continuously improving the quality and depth of our work.

# ****Comment 8:****5. Conclusion: the last sentence of topic (3) is almost the same as the sentence that appears in the last paragraph of Conclusion section. Topic (3) is described by the authors as a ‘future steps’ guideline, therefore the sentence “In summary, the RFRS-based feature selection…” does not fit there. Also, future steps were not correctly defined: “exploration of new feature extraction techniques”, an example of such new techniques could be added. Also, testing your proposed methodology in different AC contactors’ datasets is an important future step and could be added in this topic.

# ****Response:****

**We express our heartfelt gratitude for your meticulous review and valuable feedback. Your comments have prompted us to conduct an in-depth examination and necessary standardization of our article's structure, particularly in the expression of the conclusion section. In accordance with your suggestions, we have made the following revisions to the conclusion chapter:**

****1.**We have refined each paragraph of the conclusion section to ensure that they each focus on a unique perspective, avoiding repetition of content. Specifically, we have made significant adjustments to topic (3), clarifying its focus on guiding future research directions rather than summarizing the entire article.**

****2.**The description of "future steps" has been re-envisioned to be more specific and clear. We plan to continue expanding the dataset of AC contactors to enable more in-depth testing and optimization of the RFRS method.**

****3.**We have added content exploring new feature extraction techniques, providing concrete examples. For instance, we are considering visualizing data trends as image textures and employing image processing technology to capture key features, aiming to reveal more significant trend information.**

**We believe that these adjustments not only address your concerns but also make our future research path clearer and more practical. We look forward to receiving more feedback from you and are committed to continuously improving the quality and depth of our research.**

**The revised content is located in the Conclusion section on pages 15-16 of the 'Revised Article with Changes Highlighted'. (Or, see the Conclusion section on pages 18-19 in the 'Manuscript'). The following displays the modified sections in the 'Revised Article with Changes Highlighted':**

# Conclusion:This study proposes a feature selection method based on RFRS to address the issue of feature redundancy in the vibration signals of AC contactors. Through comprehensive analysis and experimental validation, the following conclusions have been drawn:

(1)This study introduces an enhancement to the traditional Random Forest algorithm by incorporating a regularization term to guide the direction of node splits and integrating recursive elimination concepts to optimize the stopping criteria, effectively reducing the dimensionality of time-frequency domain features.

(2)By comparing with three mainstream feature selection methods (Spearman's rank correlation coefficient method, the embedded method, and the filter method), and evaluating with various types of classifiers and multiple metrics, the results demonstrate that RFRS outperforms the comparative methods in overall performance. However, the superiority of RFRS is not absolute in specific contexts, and researchers must make decisions based on actual needs. For instance, when the objective is to minimize false judgments and the data is linearly separable, Spearman's rank correlation coefficient method may be more suitable.

(3)Conducting condition recognition research on AC contactors from the perspective of vibration signals still presents numerous challenges, particularly in terms of how to extract features with more pronounced trends from the vibration signals. Therefore, future work will focus on two main aspects: one is the continuous expansion of the AC contactor dataset for testing and optimizing RFRS; the other is the exploration of novel feature extraction techniques, such as transforming data trends into image textures and utilizing image processing technologies to extract key features.

# **Response to Reviewer 2**

# The paper introduces a feature selection strategy based on Regularized Random Forest with Recursive Selection applied to vibration signals of AC contactors to enhance the accuracy of their condition identification. The topic is interesting and worth investigating. However, I suggest the following revision points before the paper is considered for publishing.

We sincerely appreciate the positive evaluation and valuable recognition you have given to our research. Feature selection, often only briefly mentioned as a secondary topic in various fields of study, lacks in-depth discussion and systematic optimization. We are aware of the crucial role feature selection plays in reducing computational resource consumption and enhancing model performance, especially when dealing with large datasets.

In addressing the feature selection issue for the vibration signals of AC contactors, we have proposed a strategy based on Regularized Random Forest with Recursive Selection. This strategy not only helps to reduce the feature dimensions and improve data processing efficiency but also provides significant support for subsequent research on the state recognition of AC contactors.

We place great importance on the revision suggestions you have offered; they are immensely helpful for improving the content of our paper and elevating the quality of our research. We will carefully consider and adopt your recommendations to further refine our study.

Once again, we express our heartfelt thanks for your meticulous review and constructive feedback. We look forward to any additional guidance you can provide and pledge to continuously enhance our research to meet the standards of academic publication.

**Note:** In this revision, we have uploaded the following documents: Cover Letter, Figure, Response to Reviewers, Manuscript, and Revised Article with Changes Highlighted.

**1.Response to Reviewers:** We have adopted a dual-color style, with red font indicating our responses to the reviewers' suggestions, and blue font representing the content modified according to the suggestions.

**2.Revised Article with Changes Highlighted:** To facilitate effective re-review by the reviewers, this document retains the original format, with figures directly inserted into the text.

**3.Manuscript:** To comply with publication standards, this document has been adjusted according to the PLOS ONE style. Figures have been processed with the PACE tool and uploaded as separate files.

# ****Comment 1:**** In the abstract, there is no contextualization of the problem. Why is this development worth investigating?

# ****Response:****

# We sincerely appreciate your meticulous guidance and valuable feedback. We recognize that in our pursuit of brevity in the abstract, we failed to provide adequate context and significance of the research problem, which may affect the readers' comprehensive understanding of our work.

# In the daily operation and maintenance of AC contactors, accurate state recognition is crucial for preventing equipment failures and optimizing maintenance strategies. However, existing identification models based on time-frequency domain analysis are often troubled by the high redundancy of feature data. This redundancy not only increases the difficulty of data processing but also weakens the identification accuracy of the model. Therefore, developing an effective feature selection method in the time-frequency domain is significantly necessary and valuable for enhancing the accuracy and efficiency of AC contactor state recognition.

# Following your suggestions, we have rewritten the abstract to include a description of the research background, ensuring that the problem addressed is properly contextualized. We believe these additions will help readers better understand the significance and contributions of our research.

# Once again, we thank you for your professional review and insightful advice.

The revised content is located in the Abstract on page 1 of the 'Revised Article with Changes Highlighted'. (Or, see the Abstract on page 2 in the 'Manuscript'). The following displays the modified sections in the 'Revised Article with Changes Highlighted':

# **Abstract：**When conducting condition recognition research on AC contactor vibration signals through time-frequency analysis, the feature data exhibit a high degree of redundancy, which leads to repetitive information and hinders the accuracy of recognition. To address the redundancy issue in the features of AC contactor vibration signals, this study introduces a feature selection method based on Regularized Random Forest with Recursive Selection (RFRS). Initially, a test platform for AC contactor vibration signals was established, and time-frequency domain features of the AC contactor vibration signals were extracted. Subsequently, the traditional Random Forest (RF) was refined by optimizing its stopping criteria using the Recursive Feature Elimination approach and by incorporating a regularization coefficient during the splitting process to direct the split towards significant features. This modification not only enhances the Random Forest's capacity to leverage existing information but also introduces a bias, enabling it to favor important features. Finally, through case analysis, the proposed method effectively reduced the dimensionality of the feature set and achieved an average of 87.37% for Recall, 87.41% for F1-Score, 88.38% for Precision, and 85.74% for Accuracy. The overall performance of this method surpasses that of the three mainstream feature selection methods: Spearman's rank correlation coefficient method, the embedded method, and the filter method. This study thus provides a rather effective feature selection approach for the state recognition study of AC contactors.

# ****Comment 2:**** In the abstract, what is the contribution of the paper?

# ****Response:****

We extend our heartfelt thanks for your valuable opinions and professional guidance. Your feedback has been instrumental to us, and we have revised the abstract accordingly to more clearly present the innovation and academic contributions of our study. Here are the three main contributions of our work:

**1.**We constructed a specialized experimental platform for the vibration signals of AC contactors and detailed key parameters such as sensor models in the paper, providing reproducible experimental conditions that facilitate further research in this field.

**2.**Addressing the high redundancy issue in the vibration signals of AC contactors, we proposed a feature selection method based on Regularized Random Forest with Recursive Selection. Through empirical validation, our method demonstrates superior identification accuracy compared to existing techniques, such as Spearman's rank correlation coefficient method, the embedded method, and Filter method, which may encounter challenges when dealing with highly redundant data.

**3.**Our method introduces innovative improvements to the traditional random forest by optimizing the stopping criteria with recursive ideas and incorporating a regularization coefficient to guide the splitting process, effectively addressing the issue of unbiased feature selection in traditional random forests and enhancing the recognition of key features.

We believe that these contributions not only meet your expectations but also provide new perspectives and effective tools for the state recognition research of AC contactors. Once again, we thank you for your professional review and constructive comments.

The revised content is located in the Abstract on page 1 of the 'Revised Article with Changes Highlighted'. (Or, see the Abstract on page 2 in the 'Manuscript'). The following displays the modified sections in the 'Revised Article with Changes Highlighted':

**Abstract：**When conducting condition recognition research on AC contactor vibration signals through time-frequency analysis, the feature data exhibit a high degree of redundancy, which leads to repetitive information and hinders the accuracy of recognition. To address the redundancy issue in the features of AC contactor vibration signals, this study introduces a feature selection method based on Regularized Random Forest with Recursive Selection (RFRS). Initially, a test platform for AC contactor vibration signals was established, and time-frequency domain features of the AC contactor vibration signals were extracted. Subsequently, the traditional Random Forest (RF) was refined by optimizing its stopping criteria using the Recursive Feature Elimination approach and by incorporating a regularization coefficient during the splitting process to direct the split towards significant features. This modification not only enhances the Random Forest's capacity to leverage existing information but also introduces a bias, enabling it to favor important features. Finally, through case analysis, the proposed method effectively reduced the dimensionality of the feature set and achieved an average of 87.37% for Recall, 87.41% for F1-Score, 88.38% for Precision, and 85.74% for Accuracy. The overall performance of this method surpasses that of the three mainstream feature selection methods: Spearman's rank correlation coefficient method, the embedded method, and the filter method. This study thus provides a rather effective feature selection approach for the state recognition study of AC contactors.

# ****Comment 3:**** In the introduction, please consider reformulating or removing the phrase “It is known from statistical knowledge that the standard deviation is closely related to variance” given that “standard deviation is the square root of the variance”.

# ****Response:****

We extend our heartfelt thanks for the valuable suggestions you have offered. Our initial phrasing aimed to highlight the issue of redundancy in the feature data, thereby underscoring the importance of our research. However, after earnestly considering your feedback, we recognized that our expression did not fully align with academic standards and writing conventions.

In light of your recommendations, we have removed the original phrasing and have provided a more comprehensive discussion on the origins of redundancy and its specific impact on the feature selection process in Section 4.1, titled "Feature selection model based on RFRS." We believe these revisions not only enhance the paper's adherence to academic standards but also improve the overall flow and clarity of the presentation.

We are grateful for your expert guidance and have thoroughly revised the introduction to ensure its accuracy and compliance with scholarly writing practices.

The revised content is located in the Introduction on page 2 of the 'Revised Article with Changes Highlighted'. (Or, see the Introduction on pages 3-4 in the 'Manuscript'). The following displays the modified sections in the 'Revised Article with Changes Highlighted':

# **Introduction:**

# **......**

# Statistical analysis of vibration signal time series and spectral data can yield feature data such as amplitude, amplitude standard deviation, and amplitude variance. However, there is often a varying degree of correlation among these features; the higher the correlation, the greater the redundancy in information, ultimately leading to data redundancy that reduces both database storage space and the accuracy of condition recognition. Mainstream feature selection methods include the correlation coefficient method, embedded methods, filter methods, and recursive elimination methods. The correlation coefficient method is relatively simple, but the setting of the threshold is subjective and requires multiple subjective judgments to select the optimal features [16], hence it is inefficient on high-dimensional data sets [17-20]. The embedded method, which typically uses the random forest model as a base learner, is suitable for high-dimensional data sets and can greatly improve the efficiency of feature selection compared to the correlation coefficient method. However, due to the model's model's lack of bias, the embedded method may split nodes along non-critical features, thereby affecting the model's decision-making process [21-22]. Recursive elimination method exhaustively searches all possible feature combinations, which is conducive to finding the true key features, but it often takes a lot of time on high-dimensional data sets. Therefore, this study introduces a regularization coefficient into the random forest splitting process to guide the random forest to split in the direction of important features, and combines the recursive elimination method to optimize the stopping criterion of the random forest, achieving an organic combination of subjective and objective in the feature selection process.

# **......**

# ****Comment 4:**** In the introduction, “[19-27]” avoid using nested citation.

# ****Response:****

We extend our heartfelt thanks for pointing out the issue with our citation practices. We acknowledge that our previous method of citation may have been overly complex, not adhering to the simplicity that academic writing strives for, and potentially hindering readers' ability to trace the original literature.

Following your advice, we have thoroughly revised the introduction and reference sections. We have streamlined our citations, ensuring that each one directly and clearly points to its original source, thereby enhancing the readability and academic rigor of our paper.

We express our gratitude once again for your meticulous review and constructive feedback. We believe that with these revisions, the quality of our manuscript will be elevated to meet the standards of academic publication.

The revised content is located in the Introduction on page 2 of the 'Revised Article with Changes Highlighted'. (Or, see the Introduction on pages 3-4 in the 'Manuscript'). The following displays the modified sections in the 'Revised Article with Changes Highlighted':

**Introduction:**

**......**

Statistical analysis of vibration signal time series and spectral data can yield feature data such as amplitude, amplitude standard deviation, and amplitude variance. However, there is often a varying degree of correlation among these features; the higher the correlation, the greater the redundancy in information, ultimately leading to data redundancy that reduces both database storage space and the accuracy of condition recognition. Mainstream feature selection methods include the correlation coefficient method, embedded methods, filter methods, and recursive elimination methods. The correlation coefficient method is relatively simple, but the setting of the threshold is subjective and requires multiple subjective judgments to select the optimal features [16], hence it is inefficient on high-dimensional data sets [17-20]. The embedded method, which typically uses the random forest model as a base learner, is suitable for high-dimensional data sets and can greatly improve the efficiency of feature selection compared to the correlation coefficient method. However, due to the model's model's lack of bias, the embedded method may split nodes along non-critical features, thereby affecting the model's decision-making process [21-22]. Recursive elimination method exhaustively searches all possible feature combinations, which is conducive to finding the true key features, but it often takes a lot of time on high-dimensional data sets. Therefore, this study introduces a regularization coefficient into the random forest splitting process to guide the random forest to split in the direction of important features, and combines the recursive elimination method to optimize the stopping criterion of the random forest, achieving an organic combination of subjective and objective in the feature selection process.

**......**

# ****Comment 5:**** In equation (1), what is wr and wl? Please define all terms in all equations.

# ****Response:****

**We sincerely appreciate your meticulous guidance. We previously considered the variables *w*_R_ and *w*_L_ in** **equation (1) as intermediate terms, not of significant importance, and thus did not provide explicit definitions for them. However, with the benefit of your valuable advice, we have come to deeply recognize the importance of clearly defining all variables, which we had overlooked.**

**In response, we have re-examined every part of the paper, paying particular attention to the physical meanings and definitions of the variables in the equations. We have ensured that each variable in the text is accompanied by an accurate description of its physical significance, to facilitate a deeper understanding of the content by the readers and to enhance the transparency and readability of the paper.**

**We express our heartfelt thanks once again for your professional review and constructive comments, and we look forward to receiving more of your feedback.**

The revised content is located at equation (1) on page 4 of the 'Revised Article with Changes Highlighted'. (Or, see equation (1) on page 5 in the 'Manuscript'). The following displays the modified sections in the 'Revised Article with Changes Highlighted':

The specific process can be described by the following steps:

(1) Let the dataset be defined as *X*={(***x***_1_,*y*_1_),(***x***_2_,*y*_2_),…,(***x****_N_*,*y_N_*)}, where *N* is the number of samples. For the *j*-th sample (***x****_j_*,*y_j_*) (*j*=1,2,…,*N*), *y_j_* represents the state class (with a total of *Y* classes), and ***x****_j_* is the feature vector. When there are *F* features, ***x****_j_* is represented as (*x_j_*_,1_,*x_j_*_,2_,…,*x_j_*_,_*_F_*), where *x_j_*_,_*_f_(f*=1,2,…,*F*) denotes the value of the *f*-th feature for the *j*-th sample.

(2) Randomly extract *n*(1≦*n*≦*N*) subsets of equal size from the dataset *X*. These subsets are used to construct *n* decision tree models. For the *m*-th decision tree model (1≦*m*≦*n*), the input dataset is denoted as *X*_subset,_*_m_*, with a sample size of *N*_subset,_*_m_* and the number of features being *F*.

(3) The decision tree calculates the Gini index (*C*_Gini,_*_v_*) for the *v*-th node based on the samples of different classees at that node. It also calculates the Gini index for the left child node (*C*^L^_Gini,_*_v_*_+1_) and the right child node (*C*^R^_Gini,_*_v_*_+1_) of the subsequent node, using the difference in Gini index between the *v*-th node and the (*v*+1)-th node as the information gain to determine the direction of the decision tree split. The relevant calculation formulas are as follows:

$\left\{\begin{matrix} C_{\text{Gini,}v}=\sum_{y}^{Y} (\frac{N_{\text{subset,}m}}{N_{y}})^{2} \\ C_{\text{Gain,}v}=C_{\text{Gini,}v}-w_{\text{R}}C_{\text{Gini,}v+1}^{\text{R}}-w_{\text{L}}C_{\text{Gini,}v+1}^{\text{L}} \\ w_{\text{R}}=\frac{N_{v+1}^{\text{R}}}{N_{\text{subset,}m}} \\ w_{\text{L}}=\frac{N_{v+1}^{\text{L}}}{N_{\text{subset,}m}} \end{matrix}\right.$ (1)

In equation (1), *N_y_* represents the number of samples for state *y* at the *v*-th node, while *N*^R^*_v_*_+1_ and *N*^L^*_v_*_+1_ denote the number of samples at the right and left child nodes, respectively, of the (*v*+1)-th node. *w*_R_ and *w*_L_ represent the proportions of the samples in the right child node *N*^R^*_v_*_+1_ and the left child node *N*^L^*_v_*_+1_ relative to *N*_subset,_*_m_*, respectively.

**......**

# ****Comment 6:**** Fig. 2 is not cited in the text.

# ****Response:****

# **We sincerely appreciate your meticulous review. The oversight you pointed out indeed existed, and it was our negligence. We have now appropriately cited Fig 2 within the relevant sections of the article to ensure that all figures are clearly referenced and discussed in the text.**

# **We extend our sincere apologies for the omission in the previous version and pledge to pay closer attention to the accuracy and completeness of details in our future work.**

# **Once again, we thank you for your valuable feedback and look forward to more of your guidance.**

The revised content is located at the end of the fourth paragraph on page 5 of the 'Revised Article with Changes Highlighted'. (Or, see line 141 on page 7 in the 'Manuscript'). The following displays the modified sections in the 'Revised Article with Changes Highlighted':

**2.2 Regularized random forest with recursive selection**

**......**

It should be considered that the modified model can only more accurately calculate the importance of each feature, and the judgment of key features still necessitates the integration of subjective experience. Therefore, this study further optimizes the model by incorporating the concept of recursive feature elimination. Assuming that the features obtained from the modified model are ordered by importance from highest to lowest as *Z*={*z*_1_,*z*_2_,...,*z_F_*}, the first *k*(Where *k*≦*F*) features of set *Z* are used as the input feature matrix for the final model. The results of the final model are then assessed twice according to the elbow method. The first assessment uses overall accuracy as the metric, comparing the difference in overall accuracy between the *k*-th and (*k*+1)-th combinations.The purpose of this assessment is to identify features that significantly enhance the identification capability. The second assessment uses the recognition accuracy rates for each state of the AC contactors as the metric, with the calculation formula referring to equation (7). The significance of this assessment lies in mitigating the impact of outliers in the recognition accuracy rates for individual states. In summary, to effectively address the feature redundancy in AC contactors, this study proposes an RFRS-based feature selection method, which refines the random forest model. The operational principle is detailed in Fig 2.

**Fig.2 Schematic of the RFRS implementation principle**

# ****Comment 7:**** Regarding the figure order, I suggest introducing and citing the figure, inserting the figure in the manuscript and then discussing it. Normally, the figures appear with any discussion.

# ****Response:****

# **We sincerely appreciate your thoughtful guidance. We have realized that in the previous manuscript, our excessive pursuit of placing figures at the beginning or end of each page actually affected the smooth understanding of the paper's content by readers.**

# **Your valuable suggestions have made us aware of this issue and prompted us to re-examine the layout and citation of our figures. Following your advice, we have now adjusted our approach: we first reference the figures in the relevant paragraphs, then insert the figures, and immediately follow with discussion in the text. This sequence is more in line with conventional academic writing and helps readers better follow our arguments and analysis.**

# **We sincerely apologize for any inconvenience caused and thank you for your professional opinions. We believe that these adjustments will make our paper clearer and more comprehensible.**

# ****Comment 8:**** Fig. 3 and its font are small, please increase it to improve readability.

# ****Response:****

# **We sincerely appreciate your feedback. We have taken your suggestion regarding the font size of Fig 3 to heart and have increased the font size to ensure clarity and aid in the reader's comprehension of our research work.**

# **We apologize for any inconvenience this may have previously caused and thank you for your meticulous review and valuable suggestions. We believe these improvements will make our paper more polished in presentation and in line with academic publishing standards.**

The revised content is located in section 3.1 on page 6 of the 'Revised Article with Changes Highlighted'. (Or, see section 2.1 on pages 7-8 in the 'Manuscript'). The following displays the modified sections in the 'Revised Article with Changes Highlighted':

**3.1 Test principle**

AC contactors rely on the closing and separation actions of their moving and static contacts to manage the conduction and interruption states of the circuit, as depicted in Fig 3. When current flows through the spring coil, the moving contact is subjected to an electromagnetic repulsive force, causing it to move towards and eventually close with the static contact. At this juncture, the contactor is in a closed state, the circuit is active, and current flows freely. Upon cessation of current through the spring coil, the moving contact, influenced by the spring's pull, disengages from the static contact. At this point, the contactor is open, and the circuit is interrupted. With an increasing number of operations, the erosive effects of electrical arcs can lead to irreversible deformation of the contact surfaces. Such deformation may result in welding or mechanical interlocking of the moving and static contacts, preventing their normal separation and causing the AC contactor to malfunction, which can lead to an inability to interrupt the circuit.

**Fig.3 Schematic diagram of the working principle and fault conditions of an AC contactor**

# ****Comment 9:**** Fig. 4 and its font are small, please increase it to improve readability.

# ****Response:****

# **We sincerely appreciate your valuable feedback. We have given careful consideration to your suggestion regarding the font size in Fig 4 and have made the corresponding adjustments to increase the font size, thereby enhancing the readability of the figure. It should be noted that, due to space constraints, we have only made font adjustments to** **Fig 4A, Fig 4B, and Fig 4C; Fig 4D, which serves as a reference to Fig 2, has not undergone significant modifications. We believe these improvements will present our paper more effectively and in line with the standards of academic publishing.**

# **We extend our sincere apologies for any inconvenience the previous version may have caused to readers. At the same time, we are deeply grateful for your meticulous review and constructive suggestions.**

The revised content is located in section 3.2 on pages 6-7 of the 'Revised Article with Changes Highlighted'. (Or, see section 2.2 on pages 8-9 in the 'Manuscript'). The following displays the modified sections in the 'Revised Article with Changes Highlighted':

# **3.2 Test conditions**

**......**

Fig 4A illustrates the experimental platform for AC contactor vibration signals constructed in this study. This platform utilizes a KS76C100 vibration sensor to measure vibration signals. The sensor has an acceleration range of 60g and an output signal ratio of 100mV/g, demonstrating good measurement capabilities. Additionally, the sensor can be directly connected to a data acquisition card, which helps to minimize interference from external noise.

When the power console issues a closing command, the moving and static contacts close, generating vibration due to the impact during this closing process. Upon the issuance of an opening command, the moving and static contacts separate, and the spring mechanism connected to the contacts rebounds, leading to vibration. The sensor, mounted on the contactor housing, measures these vibrations at a sampling frequency of 1 MHz/s. The duration of the vibration signal during the opening process can be calculated using equation (8), and the signal's frequency spectrum is obtained via Fourier transform. Fig 4B illustrates the time-domain and frequency-domain diagrams of the vibration signal for a complete make-and-break operation.

**Fig.4 Test process**

# ****Comment 10:**** In Section 3.2, few details are given about the vibration time series. It should be provided and discussed the sampling frequency in Hz, the length of the time waveform, etc? Was the vibration measurement triggered with the AC contactor movement? Each measurement contains how many openings and closings of the contactor?

# ****Response:****

**Thank you for your valuable feedback. We have supplemented the section on the full-life test of the contactor in our paper with detailed information on the vibration signal time series. Specifically, Table 1 lists the conditions of the full-life test, which includes a sampling frequency of 1MHz. It should be noted that during the test, the contactor is fixed on the test bench as shown in Fig 4A, so the vibration signal measured by the vibration sensor mainly comes from the contactor itself.**

**When the power console issues a closing command, the vibration sensor starts working immediately. At this point, the moving contact and the static contact of the contactor close, and due to the impact between the contacts, a vibration signal is generated; when the power console issues an opening command, the moving contact and the static contact separate, and the movement of the moving contact also generates vibration. The vibration sensor will stop working after the vibration signal stabilizes. Therefore, each measurement includes one closing and one opening process of the contactor. Additionally, by processing the number of sampling points and the sampling frequency as shown in equation (8), we can calculate the specific time length of the vibration signal obtained in each measurement. We have further supplemented these details in the experimental section of the article.**

**We express our heartfelt gratitude for your meticulous review and constructive suggestions. We hope that this supplement meets your requirements.**

The revised content is located in section 3.2 on pages 6-7 of the 'Revised Article with Changes Highlighted'. (Or, see section 2.2 on pages 8-9 in the 'Manuscript'). The following displays the modified sections in the 'Revised Article with Changes Highlighted':

**3.2 Test conditions**

Reliability analysis of AC contactors must adhere to certain standards, meaning that feature data must be obtained under specified test conditions. Therefore, this study constructs a full-life-cycle test platform for AC contactors in accordance with the Chinese National Standard GB14048.4-2010, with some test conditions detailed in Table 1.

**Table 1. Test conditions**

| Parameter | Value | Parameter | Value |
| --- | --- | --- | --- |
| Coil voltage/V | AC 220 | Work system | AC-4 |
| Load voltage/V | 400(380) | Load current/A | 240 |
| Load type | RL | Power factor | 0.35 |
| Operating frequency/(times/h) | 300 | Sampling frequency/Hz | 1M |

Fig 4A illustrates the experimental platform for AC contactor vibration signals constructed in this study. This platform utilizes a KS76C100 vibration sensor to measure vibration signals. The sensor has an acceleration range of 60g and an output signal ratio of 100mV/g, demonstrating good measurement capabilities. Additionally, the sensor can be directly connected to a data acquisition card, which helps to minimize interference from external noise.

When the power console issues a closing command, the moving and static contacts close, generating vibration due to the impact during this closing process. Upon the issuance of an opening command, the moving and static contacts separate, and the spring mechanism connected to the contacts rebounds, leading to vibration. The sensor, mounted on the contactor housing, measures these vibrations at a sampling frequency of 1 MHz/s. The duration of the vibration signal during the opening process can be calculated using equation (8), and the signal's frequency spectrum is obtained via Fourier transform. Fig 4B illustrates the time-domain and frequency-domain diagrams of the vibration signal for a complete make-and-break operation.

$\text{Time}=\frac{N}{1\times10^{6}}$ (8)

In equation (8), *N* represents the number of sampling points.

**Fig.4 Test process**

# ****Comment 11:**** In Table 1, It is not clear if the information presented refers to the accelerometer, contactor or experiment. Please clarify it in the table and text.

# ****Response:****

# **First and foremost, we would like to express our sincere appreciation for your meticulous review and the valuable feedback you have provided. We deeply regret any inconvenience the readers may have experienced due to the unclear presentation in Table 1 of our initial submission. We recognize the necessity of obtaining the feature data of the AC contactor under specific test conditions that simulate its working environment in engineering applications to ensure the practicality and accuracy of our research.**

# **In compliance with the Chinese National Standard GB14048.4-2010, we have determined the test parameters, including operating frequency, load type, and load current, which are essential for simulating the contactor's operation in actual engineering scenarios. Our full-life test platform has been constructed according to this standard to ensure standardized operation and testing of the contactor.**

# **We have made the necessary corrections and additions to Table 1 and the related text in the main body of the paper. We have clarified that the information presented in the table specifically pertains to the test conditions for the AC contactor's feature data, rather than the accelerometer or other aspects of the experiment. We believe these revisions will enhance the clarity and professionalism of the paper, aligning it more closely with academic and engineering practice standards.**

# **We eagerly await your further feedback and are grateful for your support of our research work.**

The revised content is located in section 3.2 on pages 6-7 of the 'Revised Article with Changes Highlighted'. (Or, see section 2.2 on pages 8-9 in the 'Manuscript'). The following displays the modified sections in the 'Revised Article with Changes Highlighted':

**3.2 Test conditions**

Reliability analysis of AC contactors must adhere to certain standards, meaning that feature data must be obtained under specified test conditions. Therefore, this study constructs a full-life-cycle test platform for AC contactors in accordance with the Chinese National Standard GB14048.4-2010, with some test conditions detailed in Table 1.

**Table 1. Test conditions**

| Parameter | Value | Parameter | Value |
| --- | --- | --- | --- |
| Coil voltage/V | AC 220 | Work system | AC-4 |
| Load voltage/V | 400(380) | Load current/A | 240 |
| Load type | RL | Power factor | 0.35 |
| Operating frequency/(times/h) | 300 | Sampling frequency/Hz | 1M |

Fig 4A illustrates the experimental platform for AC contactor vibration signals constructed in this study. This platform utilizes a KS76C100 vibration sensor to measure vibration signals. The sensor has an acceleration range of 60g and an output signal ratio of 100mV/g, demonstrating good measurement capabilities. Additionally, the sensor can be directly connected to a data acquisition card, which helps to minimize interference from external noise.

When the power console issues a closing command, the moving and static contacts close, generating vibration due to the impact during this closing process. Upon the issuance of an opening command, the moving and static contacts separate, and the spring mechanism connected to the contacts rebounds, leading to vibration. The sensor, mounted on the contactor housing, measures these vibrations at a sampling frequency of 1 MHz/s. The duration of the vibration signal during the opening process can be calculated using equation (8), and the signal's frequency spectrum is obtained via Fourier transform. Fig 4B illustrates the time-domain and frequency-domain diagrams of the vibration signal for a complete make-and-break operation.

$\text{Time}=\frac{N}{1\times10^{6}}$ (8)

In equation (8), *N* represents the number of sampling points.

**Fig.4 Test process**

# ****Comment 12:**** Avoid using “frequency time waveform”, I suggest using frequency spectrum instead.

# ****Response:****

# **We extend our heartfelt thanks for your valuable feedback. We have come to fully appreciate that the use of the term "frequency-time waveform" in describing the vibration signal analysis was indeed not standardized. In accordance with your suggestion, we have replaced the relevant expressions in the manuscript with "frequency spectrum" to achieve a more professional and precise academic articulation.**

# **We believe this change will align the paper's terminology more closely with the common standards of the academic community and enhance the professionalism of the paper. We look forward to your further guidance and are grateful for your support of our research work.**

The revised content is located in section 3.3.1 on pages 7-8 of the 'Revised Article with Changes Highlighted'. (Or, see section 2.3.1 on pages 9-10 in the 'Manuscript'). The following displays the modified sections in the 'Revised Article with Changes Highlighted':

**3.3 Data** acquisition

**3.3.1 Feature extraction**

As can be inferred from the fault principles of AC contactors in Section 3.1, welding or mechanical interlocking phenomena can lead to uncertainties in the separation action. Such uncertainties are critical in determining the reliable control of the circuit. Therefore, this study focuses on the vibration signals during the separation process as the subject of research, from which time-frequency domain features are extracted. The extraction method is shown in Table 2.

**Table 2. List of time-frequency domain feature**

| Name | Formula | Description |
| --- | --- | --- |
| Time-domain peak-to-peak amplitude (T1) | $\text{T}1=\max(x_{i})-\min(x_{i})$ | The difference between the maximum and minimum signal values within a specified time frame, indicating the signal's range of variation. |
| Time-domain amplitude mean (T2) | $\text{T}2=(\sum_{i=1}^{N} x_{i})/N$ | The average value of the signal amplitudes over a specified time interval, reflecting the central tendency of the signal's amplitude. |
| Time-domain amplitude absolute mean (T3) | $\text{T}3=(\sum_{i=1}^{N} \left\vert x_{i}\right\vert)/N$ | The mean value of the absolute signal amplitudes over a specified time period, indicating the average energy of the signal. |
| Time-domain amplitude root mean square (T4) | $\text{T4=}\sqrt{(\sum_{i=1}^{N} x_{i}^{2})/N}$ | The square root of the mean of the squares of the signal amplitudes over a specified time period, a measure of the effective value of the signal's amplitude. |
| Time-domain amplitude standard deviation (T5) | $\text{T5=}\sqrt{(\sum_{i=1}^{N} (x_{i}-\text{T2})^{2})/N}$ | A measure of the amount of variation or dispersion of a set of signal amplitudes from their mean value. |
| Kurtosis factor (T6) | $\text{T6}=(\text{T2}/\text{T5})^{4}$ | A statistical measure that describes the tail weight of the amplitude distribution in comparison to a normal distribution, indicating the peakedness or flatness of the signal distribution. |
| Skewness factor (T7) | $\text{T7}=(\text{T2}/\text{T5})^{3}$ | A statistical measure that quantifies the asymmetry of the amplitude distribution around its mean, indicating the direction and degree of the data's departure from normal distribution symmetry. |
| Waveform factor (F1) | $\text{F1}=\text{T4}/\text{T3}$ | The ratio of the root mean square value to the mean value of the signal amplitude, reflecting the waveform's fluctuation characteristics. |
| Peak factor (F2) | $\text{F2}=\text{T1}/\text{T4}$ | The ratio of the peak amplitude to the root mean square value of the signal, indicating the relative magnitude of the highest peak in comparison to the average energy of the signal. |
| Impulse factor (F3) | $\text{F3}=(\text{T1}/\text{T3})^{3}$ | A measure of the impulsive characteristics of the signal, typically indicating the presence of short-duration, high-amplitude events. |
| Safety margin factor (F4) | $\text{F4}=\text{T1}/(\text{T3)}^{2}$ | A measure of the safety margin or the difference between the system's stability threshold and its current operating point, reflecting the system's resilience to disturbances. |
| Frequency-domain peak-to-peak amplitude (F5) | $\text{F5}=\max(X_{i})-\min(X_{i})$ | The difference between the maximum and minimum amplitude values in the frequency domain, indicating the range of amplitude variation across frequencies. |
| Frequency-domain amplitude mean (F6) | $\text{F6}=(\sum_{i=1}^{N} X_{i})/N$ | The average value of the amplitudes across the frequency spectrum, providing a measure of the central tendency of the signal's energy distribution. |
| Frequency-domain amplitude median (F7) | $\text{F7}=\text{median}(X_{i})$ | The median value of the spectral amplitudes when sorted in ascending order, used to mitigate the impact of extreme values on the analysis. |
| Frequency center (F8) | $\text{F8}=(\sum_{i=1}^{N} f_{i}\times X_{i})/(\sum_{i=1}^{N} X_{i})$ | The average value of the upper and lower cutoff frequencies, representing the central frequency of the signal's amplitude distribution in the frequency domain. |
| Frequency root mean square (F9) | $\text{F9}=(\sum_{i=1}^{N} f_{i}^{2}\times X_{i})/(\sum_{i=1}^{N} X_{i})$ | The square root of the mean of the squares of the amplitudes across the frequency spectrum, indicating the effective amplitude level of the signal in the frequency domain. |
| Frequency standard deviation (F10) | $\text{F10}=\sqrt{\sum_{i=1}^{N} (f_{i}-\text{F8})^{2}\times X_{i}/\sum_{i=1}^{N} X_{i}}$ | A measure of the dispersion or spread of the amplitudes across the frequency spectrum, indicating the variability of the signal's energy distribution. |

In Table 2, *x_i_* denotes the amplitude of the *i*-th sample in the time domain, *X_i_* represents the amplitude at the *i*-th frequency point, *f_i_* refers to the frequency-domain value.

# ****Comment 13:**** In equation (22), “fi refers to the frequency-domain value” explain it better.

# ****Response:****

# **Thank you for your feedback! We have taken your suggestions into account and provided a clearer explanation for the variable *f_i_*. Now, *f_i_* is explicitly defined as the frequency-domain value, which will aid readers in more accurately understanding the physical meaning expressed in the equation.**

# **We believe this improvement will further enhance the clarity and readability of the paper, ensuring that readers can comprehend our research content without any hindrance. We look forward to your further guidance and appreciate your support for our research work.**

The revised content is located in section 3.3.1, Table 2, on pages 7-8 of the 'Revised Article with Changes Highlighted'. (Or, see section 2.3.1, Table 2, on pages 9-10 in the 'Manuscript'). The following displays the modified sections in the 'Revised Article with Changes Highlighted':

**Table 2. List of time-frequency domain feature**

| Name | Formula | Description |
| --- | --- | --- |
| ...... | ...... | ...... |
| Frequency center (F8) | $\text{F8}=(\sum_{i=1}^{N} f_{i}\times X_{i})/(\sum_{i=1}^{N} X_{i})$ | The average value of the upper and lower cutoff frequencies, representing the central frequency of the signal's amplitude distribution in the frequency domain. |
| Frequency root mean square (F9) | $\text{F9}=(\sum_{i=1}^{N} f_{i}^{2}\times X_{i})/(\sum_{i=1}^{N} X_{i})$ | The square root of the mean of the squares of the amplitudes across the frequency spectrum, indicating the effective amplitude level of the signal in the frequency domain. |
| Frequency standard deviation (F10) | $\text{F10}=\sqrt{\sum_{i=1}^{N} (f_{i}-\text{F8})^{2}\times X_{i}/\sum_{i=1}^{N} X_{i}}$ | A measure of the dispersion or spread of the amplitudes across the frequency spectrum, indicating the variability of the signal's energy distribution. |

In Table 2, *x_i_* denotes the amplitude of the *i*-th sample in the time domain, *X_i_* represents the amplitude at the *i*-th frequency point, *f_i_* refers to the frequency-domain value.

# ****Comment 14:**** In Section 4.1, how training and testing datasets are selected? How many contactors have been used? How long did it take to carry out measurements? How many hours/days of experiment have been carried out? What has been done to avoid overfit of the models? What are the results during training and testing? How many samples have been considered in the training and testing datasets?

# ****Response:****

# **We sincerely appreciate the meticulous questions and valuable suggestions you have provided. Below is a detailed response to the questions you raised:**

# ****1.Data Set Selection:** In Section 4.1 of our paper, we have employed a standard seven-fold cross-validation method to divide the data set into training and testing sets. This method not only ensures the diversity and balance of the data set but also helps to reduce the risk of model overfitting. We have provided a detailed description and addition of the implementation details of the seven-fold cross-validation in Section 4.1.**

# ****2.Measurement and Experimental Time:** As shown in Table 1, our experiment operated the AC contactor at a frequency of 300 make-and-break cycles per hour. The entire process of data recording and processing took approximately five months. The actual data collection and analysis of electrical equipment is crucial for accurately reflecting its performance under actual working conditions, which is why we have invested a significant amount of time in recording various feature data throughout the entire life cycle of the electrical equipment.**

****3.Number of Contactors Utilized:** The vibration signals of AC contactors have not been extensively studied, which is a novel contribution of our research. We have documented and disclosed the setup of our experimental platform to ensure that our findings are replicable. Despite the lengthy experimental timeline, which has currently resulted in data collection from only one contactor, we have deliberately selected the CJX2-5011 model AC contactor, a widely used type, to bolster the representativeness and broad applicability of our research outcomes. We are committed to continuing our experimental work, with the objective of accumulating more data to further substantiate the approaches we have introduced.**

****3.Strategies to Prevent Overfitting:****

**(1)Introduction of Regularization Techniques: One of our main innovations is the introduction of regularization terms into the traditional random forest algorithm, which not only helps guide the splitting process but also serves as an effective penalty mechanism to prevent the model from becoming overly complex.**

**(2)Random Sampling Mechanism: Our method, an enhancement based on the traditional random forest, uses a random sampling strategy to select samples before building decision trees, which helps reduce the model's dependence on specific samples and lowers the risk of overfitting.**

**(3)Seven-fold Cross-Validation: Through this method, we ensure that the model demonstrates good generalization capabilities across different data sets.**

****4.Sample Size:** Our database contains 92,493 sets of sample data. These data, used to construct the training and testing sets through seven-fold cross-validation, ensure the stability of the model training and the accuracy of the testing. Currently, these data have been uploaded to a public database for reference and study by our academic peers.**

****5.Training and Test Set Results:** We have added the results for both the training and test sets in Section 4.3 of our paper. The results indicate that on the training set, the embedding method has the strongest learning capability; however, this method does not reduce the feature dimension. In contrast, the RFRS, Spearman's Rho, and Filter methods have all effectively reduced the feature dimensions, with RFRS showing superior learning ability over Spearman's Rho and Filter methods. On the test set, the embedding method exhibits a tendency towards overfitting with poor generalization, while RFRS demonstrates a comprehensive performance that surpasses the embedding method, Spearman's Rho, and Filter method. These results illustrate that the RFRS method proposed in this paper has significant advantages over the comparative methods.**

**We look forward to your further feedback and appreciate your ongoing support for our research work.**

The revised content regarding the training set and test set is located in section 4.1 on page 10 of the 'Revised Article with Changes Highlighted'. (Or, see section 3.1 on page 11 in the 'Manuscript'). The following displays the modified sections in the 'Revised Article with Changes Highlighted':

**4.1 Feature selection model based on RFRS**

Based on Table 2, it can be observed that time-frequency domain features share a common source of information, and some features are derived from mathematical transformations of other features. This implies that there is an overlap of information among the time-frequency domain features, which increases the complexity of data processing.

To better illustrate the application of RFRS in the domain of vibration signals for AC contactors, this section provides an analysis based on a specific example. The offline database is partitioned into a training set and a test set using a seven-fold cross-validation method, as shown in Fig 6. In each iteration, one of the seven subsets is used as the test set, while the remaining six subsets are used for training the RFRS.

**Fig.6 Principle of Sevenfold Cross-Validation**

The revised content concerning the results of the training set and test set is located in section 4.3 on pages 13-15 of the 'Revised Article with Changes Highlighted'. (Or, see section 3.3 on pages 15-18 in the 'Manuscript'). The following displays the modified sections in the 'Revised Article with Changes Highlighted':

**Table 4　Performance metrics on the training dataset**

| Method | Classifier | Recall | F1-Score | Precision | Accuracy |
| --- | --- | --- | --- | --- | --- |
| RFRS | LGBM | 94.44% | 94.44% | 94.46% | 94.44% |
|  | KNN | 92.31% | 92.29% | 92.30% | 92.29% |
|  | SVM | 89.19% | 89.14% | 89.13% | 89.14% |
|  | LR | 86.08% | 86.07% | 86.08% | 86.07% |
|  | Average Value | 90.51% | 90.49% | 90.49% | 90.49% |
| Spearman's Rho | LGBM | 93.58% | 93.58% | 93.60% | 93.58% |
|  | KNN | 91.28% | 91.26% | 91.27% | 91.28% |
|  | SVM | 88.61% | 88.57% | 88.58% | 88.61% |
|  | LR | 86.57% | 86.55% | 86.56% | 86.57% |
|  | Average Value | 90.01% | 89.99% | 90.0% | 90.01% |
| Embedded | LGBM | 95.40% | 95.40% | 95.42% | 95.40% |
|  | KNN | 92.14% | 92.14% | 92.16% | 92.14% |
|  | SVM | 90.60% | 90.60% | 90.61% | 90.60% |
|  | LR | 86.70% | 86.67% | 86.68% | 86.67% |
|  | Average Value | 91.21% | 91.20% | 91.22% | 91.20% |
| Filter | LGBM | 91.84% | 91.81% | 91.84% | 91.84% |
|  | KNN | 91.50% | 85.19% | 91.51% | 91.50% |
|  | SVM | 89.44% | 89.40% | 89.41% | 89.44% |
|  | LR | 85.74% | 85.69% | 85.67% | 85.74% |
|  | Average Value | 89.63% | 88.02% | 89.61% | 89.63% |

**Table 5　Performance metrics on the test dataset**

| Method | Classifier | Recall | F1-Score | Precision | Accuracy |
| --- | --- | --- | --- | --- | --- |
| RFRS | LGBM | 90.78% | 90.72% | 91.70% | 88.52% |
|  | KNN | 87.51% | 87.56% | 88.65% | 85.27% |
|  | SVM | 87.49% | 87.57% | 88.47% | 86.45% |
|  | LR | 83.69% | 83.78% | 84.71% | 82.73% |
|  | Average Value | 87.37% | 87.41% | 88.38% | 85.74% |
| Spearman's Rho | LGBM | 90.18% | 90.14% | 91.09% | 88.19% |
|  | KNN | 86.67% | 86.73% | 87.56% | 85.15% |
|  | SVM | 87.27% | 87.33% | 88.17% | 85.95% |
|  | LR | 84.12% | 84.20% | 85.40% | 83.33% |
|  | Average Value | 87.06% | 87.10% | 88.06% | 85.66% |
| Embedded | LGBM | 88.69% | 88.66% | 89.92% | 88.69% |
|  | KNN | 83.78% | 83.91% | 84.88% | 83.78% |
|  | SVM | 87.07% | 87.15% | 87.82% | 87.07% |
|  | LR | 83.77% | 83.83% | 84.48% | 83.77% |
|  | Average Value | 85.83% | 85.89% | 86.78% | 85.83% |
| Filter | LGBM | 87.29% | 87.35% | 87.98% | 87.29% |
|  | KNN | 85.11% | 85.19% | 85.82% | 85.11% |
|  | SVM | 86.72% | 86.76% | 87.27% | 86.72% |
|  | LR | 82.95% | 83.0% | 83.86% | 82.96% |
|  | Average Value | 85.52% | 85.58% | 86.23% | 85.52% |

Table 4 presents the performance of various classifiers on the training dataset. When considering the average scores across the four metrics, the embedded method demonstrates superior learning capability for known samples compared to other approaches. However, this method fails to effectively select key features, instead incorporating all features into the learning process. On the premise of effectively reducing feature dimensionality, RFRS outperforms Spearman's Rho and the Filter in terms of learning ability for known samples.

Table 5 illustrates the performance of various classifiers on the test dataset. An integrated analysis of Tables 4 and 5 reveals that the embedded method, hindered by feature redundancy, exhibits inferior generalizability and lower recognition capability for unknown samples compared to RFRS and Spearman's Rho. Furthermore, Table 5 demonstrates that RFRS outperforms both Spearman's Rho and the Filter across all four evaluation metrics. Specifically, in terms of Recall, RFRS achieves an average of 87.37%, slightly higher than Spearman's Rho at 87.06%, and significantly better than the Filter, which stands at 85.52%. In Precision, RFRS shows an average of 88.38%, surpassing the 88.06% of Spearman's Rho and the 86.23% of the Filter, indicating its high efficiency in reducing false positives. The F1-Score reflects RFRS's balanced capability when considering both Recall and Precision, with an average F1-Score of 87.41%, outperforming the 87.10% of Spearman's Rho and the 85.58% of the Filter. Although RFRS has a slightly lower average accuracy at 85.74% compared to Spearman's Rho at 85.66%, it still exceeds the Filter's 85.52%, with the differences being minimal, indicating that RFRS is not inferior in overall recognition performance. These results highlight the significant advantages of RFRS in the selection of time-frequency domain features for the vibration signals of AC contactors, providing an effective feature selection tool for condition recognition research.

# ****Comment 15:**** I understood that four methods have been used to compare the results: RFRS, Spearman's Rho, Embedded and Filter. I suggest describe specifically each method, considering the methods’ name used in the manuscript in a specific section. It is not clear what are the specificities of each method.

# ****Response:****

# **We extend our heartfelt thanks for the valuable feedback you have provided. In our study, we have addressed the issue of feature redundancy in AC contactors by proposing a feature selection method based on RFRS. To validate the effectiveness of our method, we have compared it with several widely-used feature selection methods, namely Spearman's Rho, Embedded, and Filter methods. We acknowledge that the original manuscript lacked a detailed introduction to these comparative methods, which may have hindered a full understanding of the advantages of the RFRS method.**

# **In light of your suggestions, we have now included a comprehensive description of each method in Section 4.2 of our paper. This addition aims to provide clarity on the characteristics of each method, facilitating a better understanding of how RFRS stands out in comparison.**

# **We eagerly await your further feedback and hope that these revisions meet with your approval. We are committed to maintaining the quality and innovation of our research work.**

# **Once again, we express our gratitude for your support and guidance.**

The revised content is located in section 4.2 on pages 11-12 of the 'Revised Article with Changes Highlighted'. (Or, see section 3.2 on pages 13-14 in the 'Manuscript'). The following displays the modified sections in the 'Revised Article with Changes Highlighted':

**4.2** Comparative methods and evaluation metrics

To elucidate the advantages of RFRS in the feature selection of AC contactor vibration signals, this study has devised three comparative methods: Spearman's rank correlation coefficient (Spearman's rho), the filter method, and the embedded method. These methods possess strong interpretability and permit the intervention of subjective experience in the decision-making process, establishing them as prevalent feature selection techniques within the field of engineering technology.

(1)Spearman's rho is a non-parametric statistical measure used to evaluate the degree of correlation between two features. The coefficient ranges from -1 to 1, with higher absolute values indicating stronger correlations and greater redundancy in the information provided by the features. The formula for calculating this coefficient is as follows:

$\rho_{\text{spearman}}=1-\frac{6\sum_{i=1}^{N} d_{i}^{2}}{N(N^{2}-1)}$ (9)

In equation (9), *d_i_* represents the measure between two features.

(2)The filter method is a variance-based feature selection technique that assesses the importance of features to a model by evaluating their variability across samples. Features with higher variance exhibit greater variability, indicating that they carry more effective information; conversely, features with variances close to zero are eliminated due to their limited contribution of useful information. The variance is calculated using the following formula:

$\sigma^{2}=\frac{1}{N-1}\sum_{i=1}^{N} (x_{i}-\overline{x})^{2}$ (10)

In equation (10),*‾x* is the sample mean of *x_i_*.

(3)The embedded method is a RF-based feature selection technique that ascertains feature importance by evaluating the contribution of each feature in reducing impurity during the construction of decision trees. The importance is calculated using the following formula:

$I_{f}=\frac{1}{T}\sum_{t=1}^{T} (C_{\text{Gain},t}^{_{\text{b}}}-C_{\text{Gain},t}^{_{\text{a}}})$ (11)

In equation (11), *I_f_* denotes the importance of the *f*-th feature, *T* is the total number of decision trees, and *C*^b^_Gain,_*_t_* and *C*^a^ _Gain,_*_t_* represent the impurity gain before and after the split in the *t*-th tree, respectively.

# ****Comment 16:**** In Section 4.3, why were the models LGBM, KNN, SVM, and LR, chosen? It is important to include a brief discussion about them and the reason to choose them.

# ****Response:****

Thank you for your feedback. We selected the models LGBM, KNN, SVM, and LR for comparison based on the following considerations:

**1.Diversity:** Our goal is to provide an effective feature selection method for contactor status identification research. Choosing different types of classifiers, such as the ensemble learning characteristics of LGBM, the distance-based classification of KNN, the margin maximization principle of SVM, and the probabilistic interpretability of LR, covers various learning paradigms. This diversity helps to evaluate the applicability and effectiveness of the feature selection method from multiple perspectives.

**2.Practicality:** These models have relatively low requirements for the volume of samples and, compared to neural network classification models, they offer stronger interpretability and a lower risk of overfitting. This makes them more practical and reliable in practical applications.

**3.Computational Efficiency:** These models are not demanding in terms of computer configuration. The computer we used is equipped with an Intel Core i5-8250U processor, 8GB of RAM, and both integrated Intel UHD Graphics 620 and discrete NVIDIA GeForce MX150 graphics cards, which is sufficient to support the training and testing of these models.

We have supplemented Section 4.2 of the paper with an introduction to these classifiers and the reasons for choosing them, hoping to provide readers with a more comprehensive understanding.

Thank you for your valuable comments and support for our work.

The revised content is located in section 4.2 on page 12 of the 'Revised Article with Changes Highlighted'. (Or, see section 3.2 on page 14 in the 'Manuscript'). The following displays the modified sections in the 'Revised Article with Changes Highlighted':

**4.2** Comparative methods and evaluation metrics

......

To comprehensively evaluate the performance enhancement of different feature selection methods in the study of condition recognition, this study employs four types of classifiers and four commonly used evaluation metrics [23-24].

The four types of classifiers are as follows:

(1)Light Gradient Boosting Machine (LGBM) is an optimized gradient boosting framework that demonstrates higher computational efficiency and superior performance compared to traditional tree models such as RF.

(2)K-Nearest Neighbors (KNN) is an instance-based learning method that classifies based on the distance between the test sample and the training samples, offering advantages in capturing complex boundaries and local structures.

(3)Support Vector Machine (SVM) is a classifier based on the principle of maximizing the margin, which achieves classification by finding the optimal hyperplane between data points and is commonly used for handling high-dimensional data and linearly separable problems.

(4)Logistic Regression (LR) is a fundamental model for multi-class problems, predicting probabilities through the output of the logistic function for classification, characterized by strong probabilistic interpretability.

The ensemble learning characteristics of LGBM, the distance-based classification of KNN, the margin maximization principle of SVM, and the probabilistic interpretability of LR encompass different learning paradigms, which can assess the applicability and effectiveness of feature selection methods from multiple perspectives.

# **......**

# ****Comment 17:**** In Section 4.3, why were the KPIs, f recall rate, F1-Score, and precision, chosen? It is important to include a brief discussion about them the reason to choose them.

# ****Response:****

# **We sincerely appreciate your valuable feedback. The selection of these KPIs, including Recall, Precision, F1-Score, and Accuracy, was intended to provide a comprehensive assessment framework for evaluating the performance of the classifiers and to gain insights into how different feature selection methods impact their effectiveness. While Recall and Precision individually reflect the sensitivity and reliability of the classifiers, they often present a trade-off scenario. The F1-Score effectively reconciles this balance, offering a holistic perspective for assessing classifier performance. Furthermore, to quickly gauge the overall identification capability of the classifiers, we have also included Accuracy as a new metric. We have supplemented Section 4.2 of our paper with a brief discussion on these evaluation metrics, elucidating the reasons for their selection and their significance in the assessment process.**

# **We look forward to your further feedback and thank you for your continued support of our research work.**

The revised content is located in section 4.2 on pages 12-13 of the 'Revised Article with Changes Highlighted'. (Or, see section 3.2 on page 15 in the 'Manuscript'). The following displays the modified sections in the 'Revised Article with Changes Highlighted':

**4.2** Comparative methods and evaluation metrics

......

The four commonly used evaluation metrics are as follows:

(1)Recall, a metric that quantifies the ratio of samples accurately classified by the model into a particular state relative to the total number of samples genuinely belonging to that state. Elevated recall signifies that the model exhibits heightened sensitivity in the detection of specific degradation states. The calculation formula for this metric is as follows:

$C_{\text{Recall}}=\frac{TP_{y}}{TP_{y}+FN_{y}}$ (12)

(2)Precision, a metric that assesses the fraction of samples that genuinely belong to a designated class within the subset that the classifier has forecasted to fall under that class. Superior precision denotes that the classifier possesses a robust level of assurance in the prognostication of specific degradation states. The calculation formula for this metric is as follows:

$C_{\text{Precision}}=\frac{TP_{y}}{TP_{y}+FP_{y}}$ (13)

(3)F1-Score, representing the harmonic mean of precision and recall, encapsulates the classifier's aggregate performance pertaining to a particular class, notably in scenarios where a compromise between precision and recall is inevitable. The calculation formula for this metric is as follows:

$C_{\text{F1-Score}}=2\times\frac{C_{\text{Precision}}\times C_{\text{Recall}}}{C_{\text{Precision}}+C_{\text{Recall}}}$ (14)

(4)Accuracy, a metric that gauges the holistic efficacy of the classifier, offers a panoramic perspective on the classifier's ability to classify across all classes effectively. The calculation formula for this metric can be found in equation (7).

Recall and precision respectively measure the sensitivity and reliability of a classifier, F1-Score is a comprehensive indicator, and accuracy provides a quick assessment of the classifier's overall recognition performance. Therefore, quantifying the performance of the classifier through these metrics can lead to a deeper understanding of the impact of different feature selection methods on the classifier.

# ****Comment 18:**** In Table 4, I suggest underlining the best results for each classification model.

# ****Response:****

# **Thank you for your valuable advice. We understand that you intend to emphasize the best performance improvements that different feature selection methods bring to various classifiers, which undoubtedly aids readers in better grasping the core value of our work. We concur with and appreciate your suggestion.**

# **However, after careful consideration, we found that Tables 4 and 5 have become quite complex after multiple revisions. If we use underlines to highlight the best results in such a context, we worry that it might increase the difficulty for readers to understand the tables. Based on this consideration, we have adopted your suggestion and made appropriate adjustments: we have used averages instead of underlines to more clearly present the results. This method not only avoids affecting the readers' understanding but also better facilitates the evaluation of the robustness and comprehensive performance of feature selection methods.**

# **We have made the corresponding modifications in Section 4.3 of the paper and ensured that these changes clearly convey our research findings.**

# **We look forward to your further feedback and thank you for your continued support of our research work.**

The revised content is located in Tables 4 and 5 on pages 13-15 of the 'Revised Article with Changes Highlighted'. (Or, see Tables 4 and 5 on pages 16-17 in the 'Manuscript'). The following displays the modified sections in the 'Revised Article with Changes Highlighted':

**Table 4　Performance metrics on the training dataset**

| Method | Classifier | Recall | F1-Score | Precision | Accuracy |
| --- | --- | --- | --- | --- | --- |
| RFRS | LGBM | 94.44% | 94.44% | 94.46% | 94.44% |
|  | KNN | 92.31% | 92.29% | 92.30% | 92.29% |
|  | SVM | 89.19% | 89.14% | 89.13% | 89.14% |
|  | LR | 86.08% | 86.07% | 86.08% | 86.07% |
|  | Average Value | 90.51% | 90.49% | 90.49% | 90.49% |
| Spearman's Rho | LGBM | 93.58% | 93.58% | 93.60% | 93.58% |
|  | KNN | 91.28% | 91.26% | 91.27% | 91.28% |
|  | SVM | 88.61% | 88.57% | 88.58% | 88.61% |
|  | LR | 86.57% | 86.55% | 86.56% | 86.57% |
|  | Average Value | 90.01% | 89.99% | 90.0% | 90.01% |
| Embedded | LGBM | 95.40% | 95.40% | 95.42% | 95.40% |
|  | KNN | 92.14% | 92.14% | 92.16% | 92.14% |
|  | SVM | 90.60% | 90.60% | 90.61% | 90.60% |
|  | LR | 86.70% | 86.67% | 86.68% | 86.67% |
|  | Average Value | 91.21% | 91.20% | 91.22% | 91.20% |
| Filter | LGBM | 91.84% | 91.81% | 91.84% | 91.84% |
|  | KNN | 91.50% | 85.19% | 91.51% | 91.50% |
|  | SVM | 89.44% | 89.40% | 89.41% | 89.44% |
|  | LR | 85.74% | 85.69% | 85.67% | 85.74% |
|  | Average Value | 89.63% | 88.02% | 89.61% | 89.63% |

**Table 5　Performance metrics on the test dataset**

| Method | Classifier | Recall | F1-Score | Precision | Accuracy |
| --- | --- | --- | --- | --- | --- |
| RFRS | LGBM | 90.78% | 90.72% | 91.70% | 88.52% |
|  | KNN | 87.51% | 87.56% | 88.65% | 85.27% |
|  | SVM | 87.49% | 87.57% | 88.47% | 86.45% |
|  | LR | 83.69% | 83.78% | 84.71% | 82.73% |
|  | Average Value | 87.37% | 87.41% | 88.38% | 85.74% |
| Spearman's Rho | LGBM | 90.18% | 90.14% | 91.09% | 88.19% |
|  | KNN | 86.67% | 86.73% | 87.56% | 85.15% |
|  | SVM | 87.27% | 87.33% | 88.17% | 85.95% |
|  | LR | 84.12% | 84.20% | 85.40% | 83.33% |
|  | Average Value | 87.06% | 87.10% | 88.06% | 85.66% |
| Embedded | LGBM | 88.69% | 88.66% | 89.92% | 88.69% |
|  | KNN | 83.78% | 83.91% | 84.88% | 83.78% |
|  | SVM | 87.07% | 87.15% | 87.82% | 87.07% |
|  | LR | 83.77% | 83.83% | 84.48% | 83.77% |
|  | Average Value | 85.83% | 85.89% | 86.78% | 85.83% |
| Filter | LGBM | 87.29% | 87.35% | 87.98% | 87.29% |
|  | KNN | 85.11% | 85.19% | 85.82% | 85.11% |
|  | SVM | 86.72% | 86.76% | 87.27% | 86.72% |
|  | LR | 82.95% | 83.0% | 83.86% | 82.96% |
|  | Average Value | 85.52% | 85.58% | 86.23% | 85.52% |

# ****Comment 19:**** In “As depicted in Table 4, the average scores for Recall, F1-Score, and Precision for RFRS are 87.37%, 87.41%, and 84.71%, respectively; for Spearman's rho, the corresponding scores are 82.06%, 87.1%, and 88.06%, respectively; and for the Filter method, the scores are 85.52%, 85.58%, and 86.23%, respectively. Comparatively, the Recall score of RFRS is 5.31% higher than that of Spearman's rho and 1.85% higher than the Filter method; its F1-Score is 0.31% higher than Spearman's rho and 1.83% higher than the Filter method; whereas its Precision score is 3.35% lower than Spearman's rho and 1.52% lower than the Filter method.”, the average values are not highlighted in the Table.

# ****Response:****

We sincerely appreciate your valuable feedback. We have realized that not displaying the average values directly in the tables, while only using them as evaluation metrics in the text, may have impeded a clear understanding of our research findings by the readers. To address this, we have revised Tables 4 and 5 to now prominently feature the average values. Your insightful suggestions are invaluable and have greatly enhanced the clarity and transparency of our work.

We thank you once again for your meticulous review and constructive comments.

The revised content is located in Tables 4 and 5 on pages 13-15 of the 'Revised Article with Changes Highlighted'. (Or, see Tables 4 and 5 on pages 16-17 in the 'Manuscript'). The following displays the modified sections in the 'Revised Article with Changes Highlighted':

**Table 4　Performance metrics on the training dataset**

| Method | Classifier | Recall | F1-Score | Precision | Accuracy |
| --- | --- | --- | --- | --- | --- |
| RFRS | LGBM | 94.44% | 94.44% | 94.46% | 94.44% |
|  | KNN | 92.31% | 92.29% | 92.30% | 92.29% |
|  | SVM | 89.19% | 89.14% | 89.13% | 89.14% |
|  | LR | 86.08% | 86.07% | 86.08% | 86.07% |
|  | Average Value | 90.51% | 90.49% | 90.49% | 90.49% |
| Spearman's Rho | LGBM | 93.58% | 93.58% | 93.60% | 93.58% |
|  | KNN | 91.28% | 91.26% | 91.27% | 91.28% |
|  | SVM | 88.61% | 88.57% | 88.58% | 88.61% |
|  | LR | 86.57% | 86.55% | 86.56% | 86.57% |
|  | Average Value | 90.01% | 89.99% | 90.0% | 90.01% |
| Embedded | LGBM | 95.40% | 95.40% | 95.42% | 95.40% |
|  | KNN | 92.14% | 92.14% | 92.16% | 92.14% |
|  | SVM | 90.60% | 90.60% | 90.61% | 90.60% |
|  | LR | 86.70% | 86.67% | 86.68% | 86.67% |
|  | Average Value | 91.21% | 91.20% | 91.22% | 91.20% |
| Filter | LGBM | 91.84% | 91.81% | 91.84% | 91.84% |
|  | KNN | 91.50% | 85.19% | 91.51% | 91.50% |
|  | SVM | 89.44% | 89.40% | 89.41% | 89.44% |
|  | LR | 85.74% | 85.69% | 85.67% | 85.74% |
|  | Average Value | 89.63% | 88.02% | 89.61% | 89.63% |

**Table 5　Performance metrics on the test dataset**

| Method | Classifier | Recall | F1-Score | Precision | Accuracy |
| --- | --- | --- | --- | --- | --- |
| RFRS | LGBM | 90.78% | 90.72% | 91.70% | 88.52% |
|  | KNN | 87.51% | 87.56% | 88.65% | 85.27% |
|  | SVM | 87.49% | 87.57% | 88.47% | 86.45% |
|  | LR | 83.69% | 83.78% | 84.71% | 82.73% |
|  | Average Value | 87.37% | 87.41% | 88.38% | 85.74% |
| Spearman's Rho | LGBM | 90.18% | 90.14% | 91.09% | 88.19% |
|  | KNN | 86.67% | 86.73% | 87.56% | 85.15% |
|  | SVM | 87.27% | 87.33% | 88.17% | 85.95% |
|  | LR | 84.12% | 84.20% | 85.40% | 83.33% |
|  | Average Value | 87.06% | 87.10% | 88.06% | 85.66% |
| Embedded | LGBM | 88.69% | 88.66% | 89.92% | 88.69% |
|  | KNN | 83.78% | 83.91% | 84.88% | 83.78% |
|  | SVM | 87.07% | 87.15% | 87.82% | 87.07% |
|  | LR | 83.77% | 83.83% | 84.48% | 83.77% |
|  | Average Value | 85.83% | 85.89% | 86.78% | 85.83% |
| Filter | LGBM | 87.29% | 87.35% | 87.98% | 87.29% |
|  | KNN | 85.11% | 85.19% | 85.82% | 85.11% |
|  | SVM | 86.72% | 86.76% | 87.27% | 86.72% |
|  | LR | 82.95% | 83.0% | 83.86% | 82.96% |
|  | Average Value | 85.52% | 85.58% | 86.23% | 85.52% |

# ****Comment 20:**** In the Conclusion, “current mainstream feature selection methods.”, please name the methods. In Conclusion, “, although the specific application scenarios still require researchers to make decisions based on actual needs.”, please be more specific.

# ****Response:****

We sincerely appreciate your valuable feedback. By comparing RFRS with three mainstream feature selection methods—Spearman's Rank Correlation Method, Embedded Method, and Filter Method—we evaluated them using various types of classifiers and multiple metrics. The results indicate that RFRS generally outperforms these comparison methods in terms of overall performance.

However, from the trade-off relationship between recall and precision, we recognize that the advantages of RFRS are not absolute in all cases. To illustrate this more concretely, we have added an example in our conclusion: when the goal is to minimize incorrect judgments and the data is linearly separable, Spearman's Rank Correlation Method may be more suitable due to its specific applicability. This underscores the need for researchers to make decisions based on actual application requirements and data characteristics when selecting a feature selection method.

We believe that by providing such detailed and specific information, readers will gain a deeper understanding of the considerations for applying different feature selection methods in various contexts, enabling them to make more appropriate choices.

We thank you once again for your meticulous review and constructive comments.

The revised content is located in the Conclusion section on pages 15-16 of the 'Revised Article with Changes Highlighted'. (Or, see the Conclusion section on pages 18-19 in the 'Manuscript'). The following displays the modified sections in the 'Revised Article with Changes Highlighted':

**5. Conclusion**

This study proposes a feature selection method based on RFRS to address the issue of feature redundancy in the vibration signals of AC contactors. Through comprehensive analysis and experimental validation, the following conclusions have been drawn:

(1)This study introduces an enhancement to the traditional Random Forest algorithm by incorporating a regularization term to guide the direction of node splits and integrating recursive elimination concepts to optimize the stopping criteria, effectively reducing the dimensionality of time-frequency domain features.

(2)By comparing with three mainstream feature selection methods (Spearman's rank correlation coefficient method, the embedded method, and the filter method), and evaluating with various types of classifiers and multiple metrics, the results demonstrate that RFRS outperforms the comparative methods in overall performance. However, the superiority of RFRS is not absolute in specific contexts, and researchers must make decisions based on actual needs. For instance, when the objective is to minimize false judgments and the data is linearly separable, Spearman's rank correlation coefficient method may be more suitable.

(3)Conducting condition recognition research on AC contactors from the perspective of vibration signals still presents numerous challenges, particularly in terms of how to extract features with more pronounced trends from the vibration signals. Therefore, future work will focus on two main aspects: one is the continuous expansion of the AC contactor dataset for testing and optimizing RFRS; the other is the exploration of novel feature extraction techniques, such as transforming data trends into image textures and utilizing image processing technologies to extract key features.
